# Differentiation between regulated and disrupted growth arrests allows tailoring of effective treatments for antibiotic persistence

**DOI:** 10.1126/sciadv.adt6577

**Published:** 2026-01-02

**Authors:** Adi Rotem, Yoav Kaplan, Orit Gefen, Irine Ronin, Alon Gutfreund, Hagai Rappeport, Raya Faigenbaum-Romm, Nitsan Naor, Elisheva Stav, Oded Agam, Nathalie Q. Balaban

**Affiliations:** Racah Institute of Physics, The Hebrew University of Jerusalem, Jerusalem 9190401, Israel.

## Abstract

Antibiotic persistence, typically attributed to dormant bacteria, is known to be a major cause of treatment failure. However, despite many years of intense research, no clear consensus on its mechanism has emerged. Here, we demonstrate that high survival under antibiotics may originate from two fundamentally different growth-arrest archetypes: either from a regulated growth arrest, leading to a protected dormant cellular state, or from a dysregulated disrupted growth arrest. Using modeling and experimental approaches including transcriptomics, microcalorimetry, and microfluidics, we unveil the characteristics and vulnerabilities of each growth-arrest archetype. In particular, disrupted bacteria show a general impairment of membrane homeostasis. This understanding resolves previous conflicting results regarding characteristics of persisters and allows tailoring treatments that target the different growth-arrested bacteria. The fundamental distinction between regulated and disrupted growth arrests should be broadly relevant for the description of cells under stress.

## Teaser

Bacteria survive antibiotics via two distinct growth arrests, each requiring a different strategy for targeting.

## INTRODUCTION

Although microorganisms spend most of their lifetime in growth arrest, the stationary phase is much less characterized than exponential growth. Pioneering work has characterized the stress responses that lead to growth arrest upon exposure to stressful conditions such as starvation (*1*–*6*). The stress response results in a protected cellular state of growth arrest, typically more tolerant to many stresses such as prolonged starvation, heat shock, oxidative stress, and various antibiotics (*7*–*13*). However, the cellular state of growth arrest in the days following the initial stress response remains poorly understood (*14*–*18*). Growth-arrested bacteria have been implicated in the failure to cure bacterial infections (*19*, *20*). Given that many antibiotics kill bacteria by targeting active growth mechanisms, nongrowing bacteria are able to persist for a very long time under these treatments (*21*). Therefore, a fundamental understanding of growth-arrest modes is key to the understanding of antibiotic persistence.

It has been suggested that natural and unnatural starvations can lead to different physiological states (*22*, *23*), indicating that persistence and tolerance may also depend on the type of starvation applied (*24*). Here, we identify two archetypes of growth-arrested bacteria: (i) a highly regulated state that follows natural starvation, where bacteria activate global stress response pathways (*25*–*30*), and (ii) a disrupted growth arrest (*31*) that follows unnatural starvation, where the growth arrest occurs due to a failure to fully activate a stress response. We find that, whereas regulated growth-arrested bacteria induce physiological changes that allow them to enter a generally protected state, the disrupted growth-arrested bacteria are dysregulated and on the verge of death. We use a model for the dynamics of the cellular state, conceptualizing regulated growth arrest as an attractor state (*32*) and disrupted growth arrest as far from an attractor state (*31*, *33*). We show that cell-to-cell variability and gene expression robustness of each archetype fits the predictions of our model. The quantitative understanding of growth-arrest archetypes lead us to unveil the loss of membrane homeostasis as a key vulnerability of disrupted bacteria obtained under various stresses and paves the way for the rational design of treatments that can selectively act on the various growth-arrested subpopulations leading to persistence.

## RESULTS

### Natural versus unnatural starvation results in two different growth-arrest modes, both equally tolerant to ampicillin

**Fig. 1.**
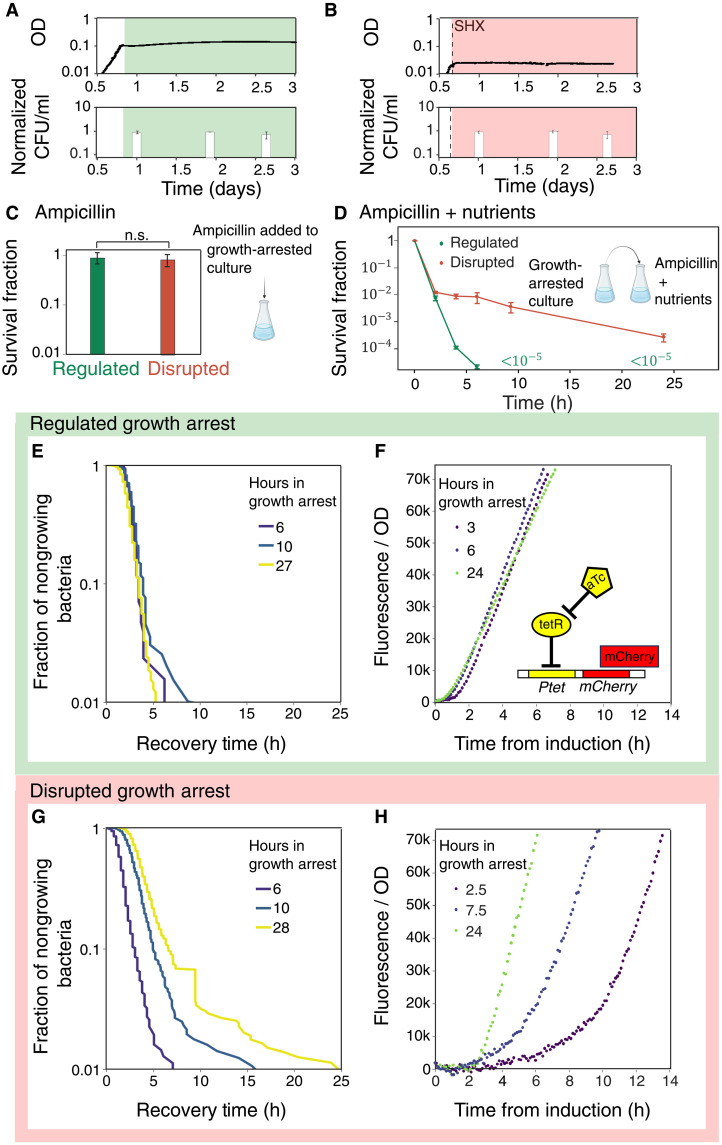
Natural and acute starvation induce two different growth arrests, both equally protected from ampicillin. (**A**) OD_600nm_ and CFU/ml over time (normalized to the maximum value) of *E. coli* KLYR grown in minimal medium, reaching natural starvation by nutrients depletion (green). (**B**) Same as (A) but starvation achieved by abrupt exposure to SHX (red) during exponential growth. (**C**) Survival of regulated and disrupted cultures arrested for 24 hours and then treated with ampicillin (50 μg/ml) for 24 hours. n.s., not significant. Error bars: SEM over biological replicates. (**D**) Survival curve of regulated and disrupted bacteria arrested for 24 hours and diluted into nutrients + ampicillin (100 μg/ml) for up to 24 hours. The surviving fraction of regulated cells fell below the detection limit (10^−5^) after 6 hours. h, hours. (**E**) Regrowth after regulated growth arrest: the fraction of bacteria remaining nongrowing despite exposure to fresh nutrients, after various starvation times (different colors) as measured using ScanLag (*99*). The dynamics do not depend on the starvation stress duration. (**F**) Fluorescence intensity per OD following induction at the regulated growth arrest. Protein production rate is constant during regulated growth arrest, independent of the starvation stress duration before induction. Inducer (aTc) was added at *t* = 0. Inset: inducible module: *P*_tet_ controls the induction of a fluorescent protein (mCherry). (**G** and **H**) Same as (E) and (F), respectively, but for disrupted bacteria. Here, the dynamics strongly depend on stress duration.

To compare different growth-arrest modes, we first drive *Escherichia coli* cultures to growth arrest using two distinct methodologies that are commonly used to study growth arrest: (i) natural starvation: the culture is allowed to progress to stationary phase by natural depletion of nutrients (*34*) (Fig. 1A), and (ii) unnatural starvation: an exponential culture is abruptly exposed to serine hydroxamate (SHX), which imposes growth arrest by mimicking an acute nutritional deprivation (*31*) (Fig. 1B). When these growth-arrested cultures are exposed to a beta-lactam antibiotic, we observe that both types of growth arrest lead to very high and similar antibiotic tolerance (Fig. 1C). However, further experiments reveal fundamental differences.

First, we study the growth arrest that occurs when amino acids are naturally depleted (Fig. 1A). This stationary phase was shown to be characterized by short recovery times upon supplementation of nutrients, regardless of the duration of starvation (*31*) (Fig. 1E). Under these starvation conditions, bacteria sharply reduce their protein production ability upon entry into stationary phase but then maintain it at a low and constant rate for days [constant activity stationary phase (CASP)] (*34*). To measure the protein production ability of bacteria at different times during CASP, we used a synthetic promoter controlling the expression of a fluorescent protein (Fig. 1F, inset), as done in (*34*). The induction curves at various time points during starvation are identical (Fig. 1F). This constant activity (constant rate of fluorescent protein production), together with the short recovery times, indicates that the starved bacteria remain prepared to quickly respond after receiving an external signal, even after days of starvation. Therefore, we term the growth arrest reached by natural depletion of nutrients “regulated growth arrest.”

In contrast to the natural starvation leading to this regulated growth arrest, we have previously shown that bacteria experiencing abrupt artificial amino acid starvation during exponential growth by exposure to SHX enter a “disrupted growth arrest” (*31*) (Fig. 1B). Unlike the regulated state, this growth arrest is characterized by a high variability of single-cell recovery times, which can span from hours to days, as well as by a strong dependence of the recovery times on the duration of the starvation (Fig. 1G). This broad heterogeneity of lag time upon recovery results in persistence to ampicillin treatment upon regrowth (Fig. 1D). To test whether the disrupted growth arrest also differs in its protein production ability, we performed the same induction dynamics experiment as in the regulated growth arrest (Fig. 1F). The fluorescence signal in the disrupted growth arrest shows the opposite to the stability observed in the regulated growth arrest: The induction curves are delayed and are strongly dependent on the duration of starvation (Fig. 1H). Counterintuitively, the fluorescent signal goes up with the duration of starvation, a hint of some dysfunction in SHX disrupted bacteria’s ability to produce protein, which is only recovered after many hours. Together, these results show that despite their similarity in ampicillin tolerance level, these two growth arrests markedly differ in their dynamics and stability.

### Increased cell-to-cell variability in the disrupted growth arrest during stress

**Fig. 2.**
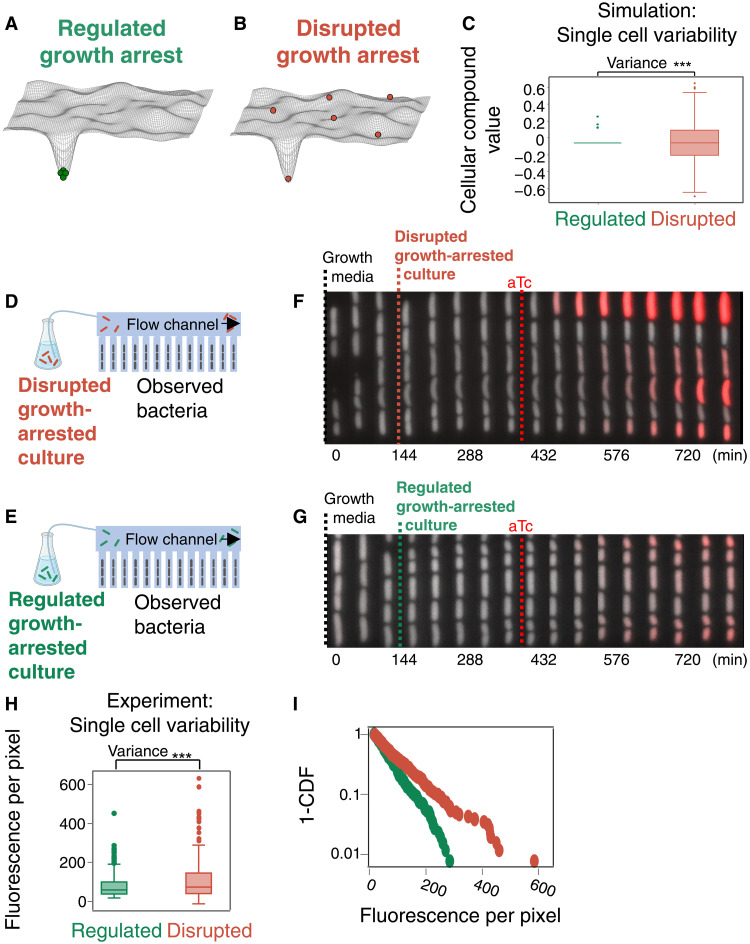
Increased cell-to-cell variability in the disrupted growth arrest. (**A** and **B**) Schematic illustration of the cellular landscape in the (A) regulated and (B) disrupted growth arrests. Cells are pictured as balls rolling in the landscape when concentrations of cellular compounds change. Regulated cells tend to be less variable as they are attracted to a specific spot. (**C**) Simulations of the variability in expression of cellular compounds using an illustrative model (see Supplementary Text). A higher variance in expression of cellular compounds is predicted for disrupted compared to regulated bacteria [Levene’s test *F*(1,19,998) = 8326, *P* < 0.005]. (**D** and **E**) Mother-machine microfluidic setup for monitoring disrupted (D) and regulated (E) growth-arrested bacteria. (**F** and **G**) Kymograph showing the time-lapse of one channel in consecutive times for disrupted (F) and regulated (G) growth arrests. Cells are supplied with growth media and then are growth-arrested for 4 hours before aTc addition. Red indicates mCherry fluorescence. (**H**) Single-cell distribution (fluorescence per pixel) under each condition 10 hours after induction. The variance is significantly larger in disrupted growth arrest [Levene’s test *F*(1,734) = 19.3, *P* < 0.005, *n* = 368]. (**I**) 1-CDF of the distributions. Box plots: horizontal line: median; box: 25 and 75% quartiles; whiskers: 1.5 interquartile range (IQR); outliers: > 1.5 IQR.

Cellular states, such as the regulated growth-arrested state, are typically described as attractors in the phenotypic landscape (*32*, *35*), pictured here as a well (Fig. 2A). However, when cells are acutely stressed, they find themselves in a rugged part of the landscape (Fig. 2B), as suggested by the unstable dynamics observed in the experiments presented in Fig. 1 (E to H). To formulate the intuitive representations shown in Fig. 2 (A and B), we simulate a random network of cellular compounds, representing abstract genes or pathways. The cellular state explores a rugged landscape with a single global attractor (see Supplementary Text). Depending on initial conditions, the cellular state may reach the attractor, representing the regulated bacteria, or the rugged part of the landscape, which represents the disrupted bacteria. Simulations of the dynamics on this landscape show that cellular compounds are predicted to be more heterogeneous at the single-cell level during disrupted growth arrest than in regulated growth arrest (Fig. 2C).

To test this prediction, we monitor cell-to-cell variability in gene induction dynamics in a microfluidics device (*36*–*38*), using the synthetic promoter controlling mCherry fluorescence depicted in the inset of Fig. 1F. To reproduce the same conditions as in batch, we flow batch growth-arrested cultures in the microfluidic flow channel (Fig. 2, D and E), as previously done (*31*, *34*, *39*). After several hours of growth arrest, the production of a fluorescent protein is induced (Fig. 2, F and G, for the disrupted and regulated cultures, respectively). We find significantly higher variability in fluorescence intensity in the disrupted than in the regulated bacteria (Fig. 2, H and I, and movies S1 and S2), in agreement with the simulation’s predictions (Fig. 2C).

In addition to the higher single-cell heterogeneity of induction levels, we find that, whereas regulated bacteria quickly respond to the inducer, disrupted bacteria require more time to reach peak protein production rate (fig. S1A). To quantify the rise time of single-cell induction curves, we fit the induction rate curves to a sigmoidal function (fig. S1B). We find that γ, the acceleration parameter of each single-cell induction curve, is significantly smaller in disrupted as compared to regulated bacteria (fig. S1C). This indicates that regulated bacteria are ready to immediately respond at full capacity to external stimulation, whereas disrupted bacteria require more time to respond.

Together, these findings support the view of the regulated growth arrest as an attractor state that reduces the single-cell variability, priming cells to respond quickly and uniformly to an external signal, whereas the disrupted bacteria are more prone to cell-to-cell variability and slower to respond.

### Impaired response to starvation in disrupted bacteria

To unveil global differences of the regulated versus the disrupted bacteria, we performed RNA sequencing (RNA-seq) on cultures during regulated and disrupted growth arrests, as well as during exponential growth. Analysis of RNA-seq often relies on the assumption that the total mRNA level remains constant under different conditions. Because the starvation response is expected to result in a reduction of the total mRNA level (*40*), we added ERCC spike-ins (*41*) to each sample, which allows estimation of absolute counts and not only relative expression (*40*, *42*). We note that all measurements reported here are above the limit of detection, as estimated using the spike-in transcripts (fig. S2).

**Fig. 3.**
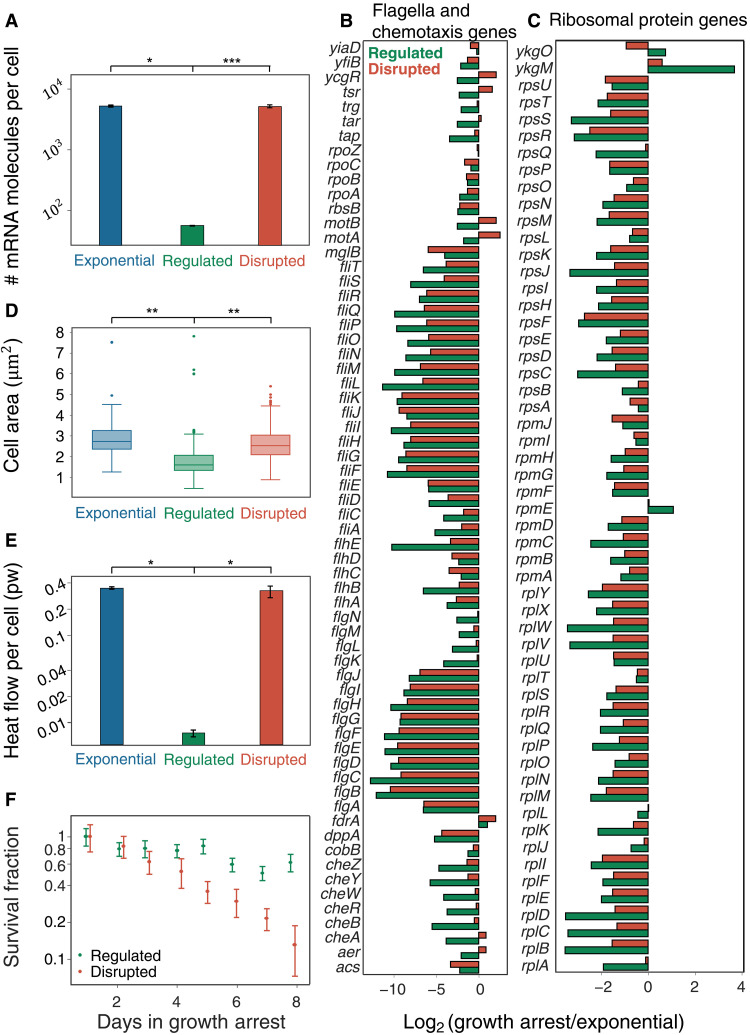
Impaired stress response in disrupted bacteria. (**A**) mRNA molecules per cell of exponentially growing bacteria as well as regulated and disrupted bacteria (Mann-Whitney *U* = 0, *P < 0.05; ***P < 0.0006). (**B**) Log_2_ fold change [LFC; DESeq2 normalization (*65*)] relative to exponential bacteria of flagella and chemotaxis genes (GO terms: GO:0009288, GO:0044781, and GO:0006935) for regulated bacteria (green) and for disrupted bacteria (red). Each row represents a gene. (**C**) Same as (B) for ribosomal protein genes (GO term: GO:0003735). (**D**) Single-cell area of exponentially growing, regulated, and disrupted bacteria (Mann-Whitney *U* = 20,924 and 49,162, **P < 0.005, *n* > 90) for the experiment described in Fig. 2 [(B) to (D)]. (**E**) Microcalorimetry measurements of the heat flow per cell of cultures in the regulated and disrupted growth arrests (Mann-Whitney *U* = 0, *P < 0.05). (**F**) Survival fraction of regulated and disrupted cultures over time during growth arrest.

We compare the regulated growth arrest to exponentially growing bacteria and find a significant reduction in the total mRNA levels (Fig. 3A), down-regulation of genes encoding for flagella and chemotaxis (Fig. 3B), and down-regulation of ribosomal protein genes (Fig. 3C), as expected in the response to starvation (*2*, *4*, *40*, *43*–*48*). However, these characteristics of the starvation stress response are significantly less pronounced in the disrupted growth arrest (Mann-Whitney test, *P* ≤ 0.05). First, the total mRNA levels in disrupted growth arrest are similar to those of exponentially growing cells (Fig. 3A), in striking contrast to the expected reduction in mRNA levels upon growth arrest. Moreover, the gene ontology (GO) analysis (*49*, *50*) revealed that, when comparing between the two growth arrests relative to exponential growth, flagellar, chemotaxis, and ribosomal protein genes showed significant differences (fig. S3 and Supplementary Text). These differences would have been overlooked if we only asked which genes increase or decrease relative to exponentially growing cells, rather than comparing the strength of the responses. Our analysis shows that disrupted cells are decreasing unnecessary pathways in a less efficient way than regulated cells.

The transcriptome profiles indicate that disrupted bacteria do activate a starvation response, but only partially. This view is reinforced by the fact that ribosomal genes up-regulated in regulated growth arrest but not in disrupted growth arrest are related to ribosome hibernation (*51*, *52*). Moreover, whereas most flagellar and chemotaxis genes are down-regulated, as expected in stationary phase (*25*, *53*), key flagellar and chemotaxis proteins are unchanged or even paradoxically up-regulated in disrupted growth arrest (see Supplementary Text). This dysregulation pattern suggests that other physiological hallmarks of the natural starvation response, such as reductive growth (*12*) and reduced metabolism (*2*, *54*), may also be impaired. In agreement with this prediction, we observe at the single-cell level that disrupted bacteria do not undergo reductive divisions, in contrast to the regulated bacteria (Fig. 3D and movies S1 and S2).

Over recent decades, a major debate in the antibiotic persistence literature has been whether decreased metabolism is a necessary condition for persistence (*54*–*59*). We thus set out to characterize the metabolism of regulated and disrupted bacteria. Using highly sensitive microcalorimetry (*60*–*63*), we measure the total amount of heat generated by each growth-arrested culture over time. In agreement with the view that regulation of growth arrest requires a controlled reduction of the metabolic rate to survive prolonged starvation (*5*, *64*), we find that the heat production rate per cell during regulated growth arrest is significantly lower compared to exponentially growing cells (Fig. 3E). In contrast, the heat production per cell in the disrupted growth arrest is much higher than in regulated growth arrest. This high metabolism is maintained for days despite the absence of growth (fig. S4).

Whereas both growth-arrested cultures survive under ampicillin treatment similarly (Fig. 1C), disrupted persisters fail to induce a proper starvation response (Fig. 3, A to D). Notably, even while growth-arrested, these cells maintain an activity level comparable to exponentially growing cells, which suggests that they may be less suited to survive long-term starvation. In agreement with this view, we find that cultures in the disrupted growth arrest start dying earlier than cultures in the regulated growth arrest (Fig. 3F). These results show that different antibiotic tolerance modes may widely differ in their metabolism and that a failure to establish an effective stress response may render disrupted bacteria more vulnerable to prolonged starvation.

### Transcriptome-wide dysregulation in the disrupted growth arrest

**Fig. 4.**
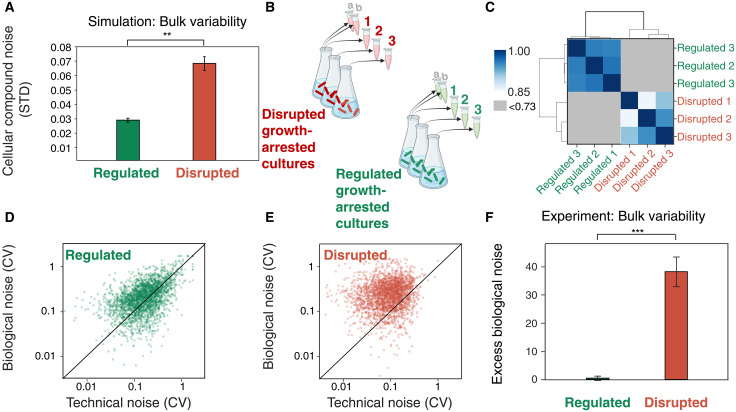
Transcriptome-wide noise in the disrupted growth arrest. (**A**) Illustrative model simulations predict higher noise in the expression of most cellular compounds in the disrupted growth arrest than in the regulated growth arrest (Mann-Whitney *U* = 235, **P < 0.005) (see Supplementary Text). (**B**) Experimental settings for measuring biological gene expression noise. Three biological replicates were grown to either regulated or disrupted growth arrest and measured by RNA-seq (“biological replicates” marked 1, 2, and 3). Two additional samples from biological replicate 1 were also collected (“technical replicates” marked a and b). (**C**) Spearman correlation between biological replicates for all protein coding genes (the same plot for the technical replicates is presented in fig. S6). (**D** and **E**) Increased genome-wide noise level in disrupted bacteria. Biological noise (CV of expression over biological samples) versus technical noise (CV of expression over technical samples) of each gene in either regulated (D) or disrupted (E) bacteria. The black line is the identity curve. (**F**) Excess biological noise: (biological variance − technical variance)/mean expression. (Mann-Whitney *U* = 1,726,993, ***P < 0.0001)

The illustrative model for the difference between regulated and disrupted growth arrests (Fig. 2, A and B) suggests that increased variability should be apparent in the disrupted growth arrest not only at the single-cell level but also at the population level (Fig. 4A and Supplementary Text). This prediction stems from the robustness of the regulated attractor state, which buffers perturbations to which the disrupted growth arrest is sensitive (fig. S17). To test this prediction, we compare the variability between biological replicates of bulk gene expression of regulated and disrupted growth-arrest cultures measured using RNA-seq. Three biological replicates were grown to either regulated or disrupted growth arrest, maintained for 24 hours, and subjected to bulk RNA extraction and sequencing (Fig. 4B). We also measured three control technical replicates to account for the measurement noise. These technical controls are obtained by splitting one starved culture of each condition into three independent RNA extractions and measurements. The expression data are then normalized by three different methods [downsampling, DESeq2 normalization (*65*), and spike-in normalization; see Materials and Methods], all leading to similar conclusions (fig. S5). First, we observe that regulated growth-arrested cultures show very high Spearman correlations between biological replicates, all above 0.97. In contrast, biological replicates in the disrupted growth arrest are significantly less correlated (Mann-Whitney, *P* < 0.05) (Fig. 4C and fig. S7B). We verified that these observations cannot be attributed to measurement error (fig. S6) as technical replicates are highly correlated (above 0.97) under both conditions.

To evaluate whether the higher variability in the disrupted cultures stems from a genome-wide effect or from a small subset of genes, we quantify the noise levels of each gene transcript. For each gene, we calculate the “biological noise” [coefficient of variation (CV) between biological replicates] and compare it to the “technical noise” (CV between technical replicates). We quantify the percent of noisy genes, for which the biological noise significantly exceeds the technical noise. In the regulated growth arrest (Fig. 4D), only 7% of genes are noisy, whereas in the disrupted growth arrest (Fig. 4E), 32% of genes are noisy (see Materials and Methods for test, *P* < 0.001). Moreover, the mean variability of the transcripts is significantly higher in disrupted growth arrest (Fig. 4F). This is quantified by the excess biological noise defined as the difference between biological and technical variance, normalized by the mean expression. This increased variability agrees with the simulation predictions presented in Fig. 4A. We note that the higher biological replicate noise was reproducible in an independent repeat of the full experiment (fig. S7), not restricted to genes with lower expression levels (fig. S8), and was independent of the normalization method (fig. S5) as well as of the noise metric (fig. S9). The variable genes in the disrupted growth arrest are significantly associated with bacterial chemotaxis and flagellar assembly, whereas no association was found between variable genes in the regulated growth arrest (see Supplementary Text).

Biological replicates of disrupted growth arrest show wide transcriptional variability, whereas regulated growth arrest is robust and consistent. This supports the view that regulated arrest represents a stable attractor state, whereas disrupted arrest reflects a random exploration of the state space (Fig. 2, A and B, and fig. S17).

### Dysregulation leads to increased vulnerability, whereas regulation protects growth-arrested bacteria

**Fig. 5.**
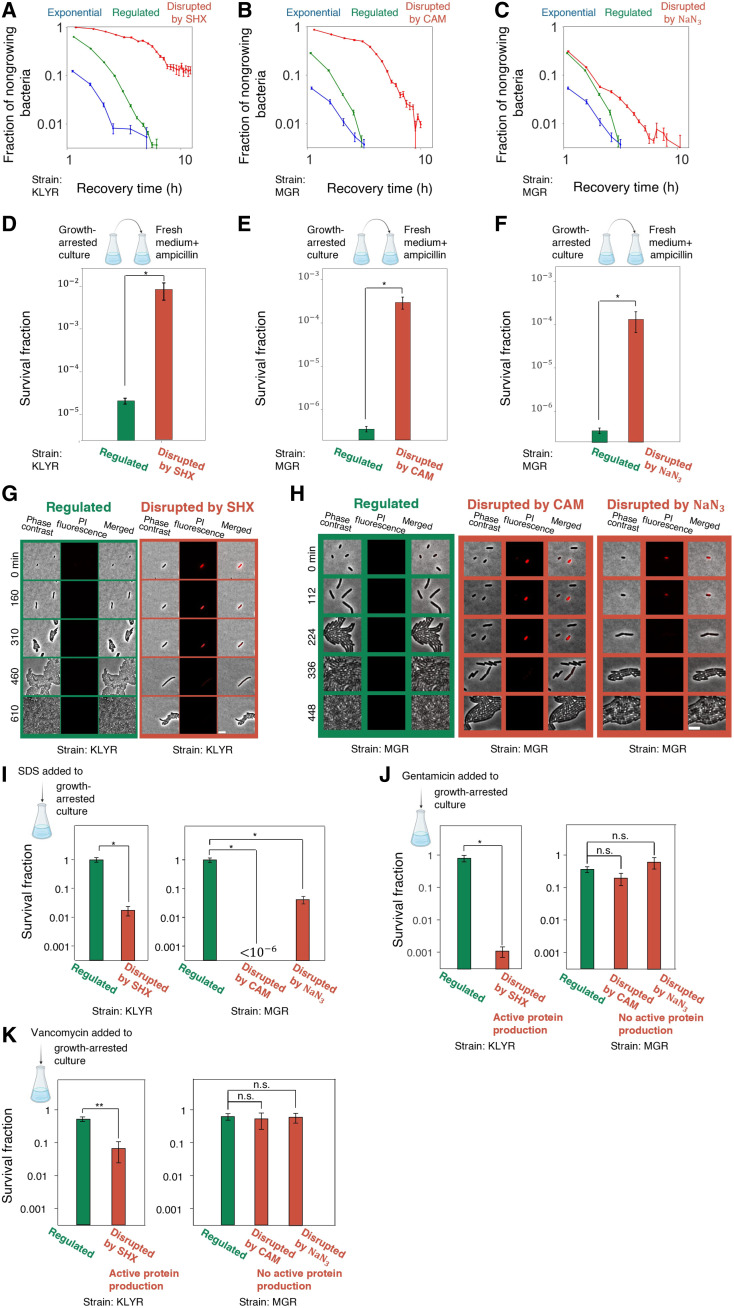
Cells disrupted by various agents have more permeable membranes and are more susceptible to SDS treatment than regulated cells. (**A** to **C**) Fraction of nongrowing bacteria upon regrowth from exponential phase (blue), regulated growth arrest (green), or disrupted growth arrest (red), measured using ScanLag (*99*); error bars are SEM over all bacteria within 0.5-hour time bins on at least three biological replicates. (A) Disruption by SHX (0.5 mg/ml). [(B) and (C)] Disrupted growth arrest induced by either (B) CAM (25 μg/ml) or (C) NaN_3_ (10.5 mM). The same plots for regulated and exponential cells are reproduced in (B) and (C). (**D** to **F**) Survival fraction of cultures that were in regulated or disrupted growth arrest and then diluted into fresh medium with ampicillin (100 μg/ml, 6 hours) where disruption was done by (D) SHX, (E) CAM, or (F) NaN_3_. (**G**) Time-lapse microscopy of PI-stained growth-arrested single cells. Cells were either grown to regulated growth arrest or disrupted by SHX for 24 hours and transferred to growth conditions (without PI) under the microscope. Regulated bacteria show significantly lower PI fluorescence, whereas disrupted bacteria may show high PI signal, which disappears upon regrowth. (**H**) Same as in (G), for growth arrest by either CAM or NaN_3_ for 24 hours. (**I**) Survival of regulated and disrupted cultures after a treatment with SDS (1%) for 24 hours. This sub-MIC SDS concentration kills only disrupted bacteria (fig. S13). (**J** and **K**) Treatment with (J) gentamicin (5.2 μg/ml) or (K) vancomycin (1500 μg/ml) for 24 hours during growth arrest is only effective against disrupted conditions with active protein production (Mann-Whitney, *P ≤ 0.05; **P ≤ 0.005).

Thus far, we have induced a disrupted growth arrest by applying abrupt serine starvation. We now include two other treatments, namely, chloramphenicol (CAM), which blocks protein synthesis, and sodium azide (NaN_3_), which depletes ATP (adenosine triphosphate) (*66*). These have very different targets but also result in a disrupted growth arrest, as identified by the characteristic variable lag times upon regrowth (*31*) (Fig. 5, A to C). Figure 5 (D to F) shows that a treatment of ampicillin together with nutrients enables to reduce the survival of regulated cells by orders of magnitude [as suggested in (*55*)], whereas the same strategy is much less effective against disrupted cells. This decreased killing of disrupted growth-arrested bacteria is due to the global dysregulation of their cellular network, which delays their regrowth (Fig. 5, A to C), despite the influx of nutrients. This shows that, to eradicate both growth-arrest types, it is necessary to take into account their individual characteristics.

The understanding that disrupted growth arrest causes global dysregulation of the cellular network suggests that homeostasis may be altered in those cells, regardless of the disrupting treatment. In particular, we hypothesized that the membrane, which requires constant repair, may be affected (*67*, *68*). To test whether the membranes of disrupted bacteria are impaired, we measured the propidium iodide (PI) uptake of single bacteria. PI is typically used as an indicator of dead cells (*69*) but may also measure the membrane permeability in live cells (*70*–*72*). We find that membrane damage, as measured by PI intake, is only apparent in disrupted cells and is significantly lower in regulated cells (fig. S10). Using microscopy, we observe that disrupted cells, under the three different conditions leading to disruption, may show PI permeability but are not dead and may regrow when the stress is removed (Fig. 5, G and H, and movies S3 and S4). To further test membrane vulnerability, we used a bona fide membrane targeting agent SDS (*73*). We find that cells that are disrupted by either SHX, CAM, or NaN_3_ are hypersensitive to SDS, when added during growth arrest (Fig. 5I), even at a concentration below the minimum inhibitory concentration (MIC) (fig. S12). This shows that, regardless of the specific mode of disruption, disrupted cells have more permeable membranes than regulated cells.

The results above show that the general understanding that growth arrest protects bacteria against many stresses may be true only for the regulated growth arrest. All disrupted conditions resulted in a higher sensitivity to some of the stresses that we studied (Fig. 5, I to K).

As illustrated in Fig. 2B, the disrupted growth arrest is not a specific region of the cellular phenotypic space and therefore we avoid calling it a “state.” Therefore, the specific manifestations of disruption may vary. For example, we observe that the ability to produce protein is maintained in cells treated by SHX (Fig. 1H) but not in cells treated with CAM (*73*) or NaN_3_ under our conditions (fig. S11). As vancomycin and gentamycin require active protein synthesis to kill cells (*55*, *74*), these treatments are therefore effective against SHX treated but not against CAM-treated or NaN_3_-treated cells (Fig. 5, J to K). This shows that, whereas all conditions leading to disruption that we have tested lead to impaired membrane, other manifestations of dysregulation under each disrupted condition may vary.

CAM inhibits protein synthesis, NaN_3_ disrupts respiration, and SHX triggers serine starvation—yet despite their distinct mechanisms, all three lead to a common outcome: a disrupted growth arrest characterized by heterogeneous recovery times, membrane permeability, and hypersensitivity (see fig. S12). Thus, the distinction between disrupted and regulated growth arrests is functionally consequential: Disruption results in membrane damage and a range of condition-specific physiology, whereas regulated growth arrest confers broad protection. This insight provides a conceptual and practical framework for tailoring treatments to eradicate the various types of persistent and tolerant bacteria.

## DISCUSSION

We have shown that growth-arrested bacteria, which exhibit similar tolerance levels to ampicillin treatment, may be in fundamentally distinct cellular physiologies. Regulated growth-arrested bacteria activate a full starvation stress response, significantly reducing mRNA levels and metabolism by orders of magnitude when compared to exponentially growing cells, reaching a dormant state that is robust against perturbation. In contrast, disrupted growth-arrested bacteria, identified by their physical aging dynamics (*31*), elicit an impaired stress response, leading to dysregulation. This dysregulation causes disrupted bacteria to be highly variable, whereas regulated bacteria are not, challenging the conventional view that stress invariably leads to increased variability (*75*–*77*).

The specific observables that display variability differ depending on the disrupting agent. For example, protein production is heterogeneous in bacteria disrupted by SHX, whereas bacteria disrupted by CAM or NaN_3_ fail to produce protein altogether. In this work, we demonstrated that SHX disruption leads to variability across many observables and that lag times are variable under the other disrupting conditions as well. Future studies using additional observables that can be evaluated across the different conditions are expected to reveal additional variability. The key distinction is that regulated growth arrest represents a well-defined state in the bacterial state space (Fig. 2A), whereas disrupted growth arrest reflects the absence of a defined state, with each bacterium instead randomly exploring a different part of the state space (Fig. 2B).

The distinction between growth arrest archetypes allows to identify drugs and protocols that can differentially target each type. Regulated bacteria start to grow promptly upon addition of nutrients, making them sensitive to antibiotics supplied with nutrients. In contrast, the dysregulation of disrupted bacteria delays their regrowth upon addition of nutrients, making this strategy less effective. However, dysregulation also results in loss of homeostasis and increased membrane permeability, which can be exploited to target them using membrane targeting agents. In a sense, despite their resilience to antibiotic treatments that target active growth mechanisms, disrupted bacteria are on the verge of death, and the way to eradicate them is to push them further along that direction. Exploiting this vulnerability, we found conditions that very effectively kill the disrupted persisters while not affecting the regulated bacteria.

The distinction between growth-arrest modes settles conflicting results concerning the level of metabolic activity of persister bacteria (*54*–*59*, *78*). We show that bacterial cultures with orders of magnitude difference in their heat production may have similar beta-lactam tolerance levels. Regulated bacteria show a very low metabolism that is reminiscent of spores (*22*). Moreover, both regulated growth-arrested bacteria and spores are resilient to stresses (*79*) and are able to resume growth quickly (*80*) and uniformly (*81*). In contrast, bacteria disrupted by SHX have a high metabolism that may be linked to futile cycles, which arise from such dysregulation, and have been previously linked to antibiotic persistence (*78*).

The two very different archetypes of tolerance explain the difficulty in identifying the “tolerome” (*82*), i.e., specific genes linked to high survival under antibiotics unrelated to resistance (*83*–*85*). Whereas genetic screens performed in the regulated growth arrest may uncover specific pathways, screens performed on the disrupted cultures are bound to fail. This is because the disrupted growth arrest arises from global dysregulation and not because of the specific activation of a tolerance pathway (*86*, *87*). Moreover, the disrupted growth arrest is not a “state” because disrupted bacteria are scattered across the cellular state space, in contrast to the regulated state that is confined to an attractor. Paraphrasing the Anna Karenina principle, “All regulated bacteria are alike, whereas each disrupted bacterium is unregulated in its own way.” This understanding holds also for disrupted populations attained by different disrupting conditions. The conclusion is that it is crucial to identify which growth-arrest mode is under study when searching for tolerance genes in a given experimental context. We provide here several indicators of disruption that may help distinguish between the two archetypes in future experiments. Disrupted persisters are characterized by long lag times, high cell-to-cell variability in their regrowth dynamics, strong dependence of lag on stress duration, as well as partial loss of membrane homeostasis.

More generally, the identification of a disrupted mode of growth arrest and its approximation by a model of random network dynamics should be relevant to other cellular systems under stress. Cancer persisters, for example, were shown to be growth-arrested for extended periods of time under drugs (*88*, *89*) and, if disrupted, may be identified with indicators similar to the ones proposed here. The fundamental understanding of growth-arrest modes offers an alternative viewpoint on cells under stress, which should lead to previously unknown avenues for more effective treatments against drug persistence.

## MATERIALS AND METHODS

### Strains and plasmids

The ancestral strain, *E. coli* KLY (*82*), was constructed by P1 transduction of the *yfp-cam*^R^ cassette from MRR (*90*) into KL16 (*91*). The *yfp-cam*^R^ cassette constitutively produces yellow fluorescent protein (YFP) under the *P_R_* promoter. To construct the KLYR strain P1_vir_ lysate of *E. coli* DH5αZ1 [obtained from H. Bujard (*92*)] was used with spectinomycin resistance selection to transduce *lacI^q^* module and *tetR* into the KLY background under constitutive promoters (*31*). The *relA1* (*93*) and *spoT1* (*94*) mutations of KLYR confer a relaxed stringent response phenotype (*94*, *95*). This strain suffers from acute stress under abrupt SHX treatment (*31*). All experiments were done on the KLYR + pZA21mCherry plasmid (*96*), unless stated otherwise. The plasmid was used for fluorescence induction measurements under the *P_L_*tetO1 promoter (*92*) (see Fig. 1E, inset).

To construct *E. coli* MGR, a P1_vir_ lysate of DH5αZ1 [obtained from H. Bujard (*92*)] was used to transduce MG1655 into MGR under a spectinomycin resistance screen to move the *lacI* and *tetR* genes into the MG1655 background under constitutive promoters (*97*).

MG1655/pZA21RmCherry is an *E. coli* MG1655 with the plasmid pZA21RmCherry. This plasmid was constructed by cloning *tetR* after *kan^R^* by transcriptional fusion in the pZA21mCherry plasmid (*31*, *34*).

To construct *E. coli* MG1655intRmCH, the λ red method (*98*) was used to transduce the *tet^R^-kan^R^-tetO-mCherry* module into the *intS* genomic region of MG1655. The module was amplified from the pZA21RmCherry plasmid (*31*).

### Biological replicates

Bacteria were streaked from frozen stock on an LB plate and grown for 15 hours at 37°C. Single colonies (biological replicates) were then picked and grown in 1 ml of LB with kanamycin (30 μg/ml; Sigma-Aldrich) at 37°C with shaking for 21 hours. Each biological replicate was then divided into small aliquots and frozen at −80°C with 15% glycerol.

### Growth and growth-arrest conditions

Bacteria were diluted 1:10^6^ from a frozen aliquot into fresh medium (M9, Sigma-Aldrich) with supplemented 0.1% Casamino acids and kanamycin (30 μg/ml; Sigma-Aldrich) for *E. coli* KLYR or without antibiotics for *E. coli* MGR. Bacteria were grown at 32°C with shaking. This temperature was chosen to reduce evaporation of the liquid medium. For RNA-seq and microfluidics experiments, growth was done in 60-ml cultures in a 250-ml Erlenmeyer flask. For all other experiments, growth was done in 96-well plates with shaking (orbital with 2.5-mm amplitude) in a plate reader with 180 μl in each well (Infinite M200 Pro, Tecan or Infinite F200 Pro, Tecan), unless stated otherwise.

To reach the regulated growth arrest, cultures were allowed to grow until nutrients were naturally depleted. To induce the disrupted growth arrest, cells were grown to exponential phase (~13 generations from the initial inoculation, when cells reach around 10^7^ cell/ml), either SHX (0.5 mg/ml), NaN_3_ (10.5 mM), or CAM (25 μg/ml) was added. If not stated otherwise, SHX was used to induce a disrupted growth arrest. Also, the time spent in growth arrest was 24 hours for regulated cells, cells disrupted by SHX and cells disrupted by CAM, and 48 hours for cells disrupted by NaN_3_, unless stated otherwise.

### OD and fluorescence measurement of bulk cultures

Bacteria were grown and measured in a plate reader (Infinite F200 Pro, Tecan), using 96-well plates. Measurements were taken as optical density (OD) (630 nm) and mCherry fluorescence (excitation: 530 nm, emission: 635 nm), unless stated otherwise.

### mCherry induction from *P*_tet_

A stock of aTc (Sigma-Aldrich) was prepared [(500 μg/ml in (double-distilled water (DDW)]. Induction was done by adding aTc from the stock to a final concentration of 0.2 μg/ml in the media, in either bulk or in the flow channel of the microfluidics device. The results in Fig. 1 (F and H) show a typical measurement for a biological replicate.

### Recovery time measurements using ScanLag

A sample of the culture was diluted and plated on M9 agar plates supplemented with either 1% Casamino acids and kanamycin (22 μg/ml; Sigma-Aldrich) for *E. coli* KLYR or without antibiotics for *E. coli* MGR. Plates were placed on automated scanners [ScanLag (*99*)] in an incubator at 32°C and imaged every 15 min. The images were analyzed using ScanLag, and the appearance time of each colony was measured. The results in Figs. 1 (E to G) and 5 (A to C) are shown for a typical biological replicate.

The fast diffusion of SHX out of *E. coli* cells was shown in (*100*). In all assays, the disrupting chemical, such as SHX, is thoroughly washed. We follow the protocol of Kaplan *et al.* (*31*), where it was demonstrated that SHX itself does not affect the dynamics posttreatment. No delays in recovery were observed when the same concentration of SHX was added gradually, when it was added at regulated growth arrest, or in the absence of SHX (*31*).

### Microscope and imaging setup

Microscopy was done using a Nikon Ti2E inverted fluorescence microscope system, ×100 NA (numerical aperture) 1.45 oil objective with phase-contrast, automated stage and shutters, and automated focus hardware [Perfect Focus System (PFS)]. Temperature was controlled using a cage incubator (Okolab). The control of the microscope, stage, shutters, camera, PFS, and image acquisition were done using Micro-Manager open-source microscopy software (*101*). Images were acquired with a sCMOS digital camera (Orca Flash 4.0v3, Hamamatsu). YFP imaging: Spectra X (Lumencor), EX filter 500/24 EM 542/27 (Semrock). Red fluorescence imaging (used in PI staining and mCherry induction assays): Spectra X (Lumencor), EX filter 562/40 EM 640/75 (Semrock).

### Microfluidics observations

The microfluidic mother-machine devices molds were kindly provided by the Paulsson laboratory (*102*). Polydimethylsiloxane (PDMS; Sylgard 184, Dow Corning) was mixed in a ratio of 10:1 part A to part B, poured on an epoxy mold (EasyCast clear casting epoxy), and cured at 60°C for 6 hours. The devices were made adherent by plasma treatment to #1.5 60 mm by 48 mm glass slides. To avoid oscillations in the PFS stabilization, the glass slide was taped to a custom-made aluminum holder.

Bacteria were first grown to regulated growth arrest and introduced into the growth channels by putting 4 to 6 μl of culture into the channel and centrifuging at 2000 to 3000*g* for ~1 hour at 10°C (Eppendorf centrifuge 5810R). Following the procedure described in (*36*), a mixture of salmon sperm DNA (10 mg/ml) and BSA (bovine serum albumin; 10 mg/ml, at a ratio 1:3) was used to passivate the device before loading the cells.

To achieve growth conditions in the microfluidics device, we supplied fresh media as described in “Growth and growth-arrest conditions.” Constant flow of medium (10 μl/min) in the device was achieved with a syringe pump (4002x, New Era). Temperature was controlled at 32°C. Bacteria were allowed to grow in the growth channels for a few hours before growth arrest. All bacteria performed at least two divisions before growth arrest. To induce growth-arrest conditions in the growth channels, we supplied either a regulated or a disrupted growth-arrested culture into the flow channel. The background regulated and disrupted cultures were grown as described in “Growth and growth-arrest conditions.” Under both growth arrest conditions bacteria in the growth channel stopped growing soon after introducing the growth-arrested cultures into the flow channel. Flow of the growth-arrested culture in the main microfluidic channel ensures reproducing the conditions in the growth-arrested bulk cultures (*34*). After 4 hours in growth arrest, the culture in the flow channel was supplemented with aTc as described in “mCherry induction from *P*_tet_.”

Image analysis was done using FIJI (*103*). Cell outlines were detected using YFP as the KLYR strain constitutively produces YFP (see “Strains and plasmids”). The fluorescence per pixel of each cell was calculated as the mCherry fluorescence summed over the area enclosed by the cell outline (where the baseline for each cell was subtracted) and divided by the area of the cell in pixels. The cell area presented in Fig. 3D is the cell area converted from pixels to micrometers.

The single-cell fluorescence intensity curves were fitted with a sigmoidal derivative, as described in the Supplementary Materials (see fig. S1).

### RNA-seq experiments

Each biological replicate was inoculated from a frozen aliquot of a different biological replicate as described in “Biological replicates.” Cultures were grown to regulated or disrupted growth arrest as described in “Growth and growth-arrest conditions.” “Technical replicates” are multiple samples taken from the same culture. They separately undergo RNA extraction, and all downstream processing and measurements. Samples of exponential growth were collected just before SHX was added. Cultures were kept in growth arrest for 24 hours before sample collection. The RNA-seq experiment was repeated twice (Supplementary Methods). The raw data are available at ArrayExpress (accession numbers E-MTAB-14335 and E-MTAB-14332).

#### Sample collection for RNA-seq

Samples of 1.8 ml were collected from each biological replicate. Each sample was spun down for 5 min at 5000*g* and 4°C. After spin down, the excess fluid was discarded and the pellet resuspended in 50 μl of TE buffer [10 mM tris-HCl (pH 7.5) and 1 mM EDTA], moved into a precooled Eppendorf with 5 μl of Lysozyme in RNase-free water (9 mg/ml; Sigma-Aldrich), quickly thrown into liquid nitrogen, and stored at −80°C.

#### RNA extraction

The frozen samples were subjected to two cycles of thawing at 37°C and refreezing in liquid nitrogen. Next, the samples were resuspended thoroughly to homogenization with 1 ml of TriReagent (prewarmed to room temperature) and incubated for 10 min at room temperature. Chloroform (200 μl) was added, and the tubes content was mixed by inversion for 15 s. The samples were incubated for 10 min at room temperature, centrifuged (14,000*g*, 10 min at 4°C), and the upper phase was collected and transferred into new Eppendorf tubes. For RNA precipitation, 500 μl of isopropanol was added, and the tube contents were mixed thoroughly by inversion and incubated for 10 min at room temperature. The tubes were centrifuged (14,000*g*, 15 min at 4°C), and the supernatant was discarded. The pellets were washed twice by addition of 1 ml of freshly made 75% (v/v) ethanol (made with diethyl pyrocarbonate–treated water and kept on ice), followed by centrifugation (14,000*g* for 5 min at 4°C) and removal of the supernatant. Next, samples were spun down for 3 min at room temperature, and the excess fluid was discarded. Pellets were dried by leaving the tubes open for 10 to 15 min at room temperature and then resuspended in 50 μl of RNase-free water and stored at −80°C. RNA quality and concentration were assessed by Nanodrop (Thermo Fisher Scientific) and by Bioanalyzer (Agilent).

#### Adding ERCC spike-ins to the RNA samples

A fixed amount of ERCC spike-ins (Thermo Fisher Scientific) (*41*) was then added per total RNA (1.3 μl of mix1 1:40 dilution to either 1 or 0.1 μg of total RNA in RNase-free water, depending on the experiment). The spike-ins were added before rRNA depletion.

#### rRNA depletion, library construction, and RNA-seq

rRNA depletion, library preparation, and RNA-seq were done by the center for genomic technologies, the Institute of Life Sciences, the Hebrew University of Jerusalem. rRNA depletion was done using the NEBNext rRNA Depletion Kit for bacteria. Library preparation was done using the NEBNext Ultra II Directional RNA Library Prep Kit. RNA-seq was done using either NextSeq 500 or NextSeq 2000 (Illumina).

#### RNA-seq data analysis

Read trimming was done using Cutadapt (*104*) (with parameters: -e 0.2 -m 15 -O 1 -q 20). Mapping was done using bowtie2 (*105*) (with parameters: --local --very-sensitive-local). The raw reads were mapped to the genome available at CP008801.1, with minor changes as described in the Zenodo repository (https://doi.org/10.5281/zenodo.15582541). Expression counting was done using htseq-count (*106*) (with parameters: stranded option, --nonunique none).

#### Normalization methods for gene expression relative to total sample expression

To calculate the downsampled raw reads (table S4), the total number of raw reads was calculated for each sample, and the minimum total reads across all samples was identified (excluding ERCC transcripts). Subsequently, all samples were randomly subsampled to match this minimum count. If all samples had <50 reads for a certain transcript, the transcript was filtered out (we note that the downsampling was done for the two growth-arrested states of each experiment together and for the exponential samples separately). Noncoding transcripts were filtered out. The data presented in Fig. 4 (C to F) and figs. S6 to S9 are normalized by this method. This avoids biases that may be due to differences in the total number of reads.

RPK (reads per kilo base pair) was calculated on the downsampled raw reads. RPK normalized data are used in fig. S5 (C and D) (note that the RPK calculated for ERCC spike-in normalization was calculated on the raw reads without downsampling).

DESeq2 differential gene expression (table S3) and DESeq2 normalization were done using the default parameters for all samples under each condition separately. The differential gene expression calculated by DESeq2 is used in Fig. 3 (B and C) and fig. S3 (A and B). Note that, in fig. S5, we only use the normalization step of DESeq2.

To get an estimate of the absolute number of molecules per cell (table S2), we used ERCC spike-ins (*41*). We used the method recommended by Thermo Fisher Scientific, with minor changes as described in Supplementary Methods.

#### Estimating the total mRNA counts under each condition

We used the ERCC-normalized data for this estimate, without the total count correction (see “Absolute gene expression normalization using ERCC spike-ins” in the Supplementary Materials). We use all samples from both experiment 1 and experiment 2 (“RNA-seq experiments”), totaling in seven biological replicates for each growth arrest and three biological replicates for the growth condition. We filter the genes for protein coding genes and sum over the number of molecules in each sample. For biological replicates that had technical replicates, we average over the technical replicates to get the value of the biological replicate. Figure 3A shows the mean and SEM over biological replicates.

#### Significance test of biological replicate correlation in RNA-seq experiments

The Spearman correlation between all pairs of biological replicates under the same condition and the same experiment was calculated. The correlation was calculated for all protein coding genes, normalized by downsampling. Then, a Mann-Whitney test evaluated whether the correlations between all pairs of regulated state samples are stochastically lower than the correlations between all pairs of disrupted samples.

#### Calculating the biological and technical noise of gene expression

We used three measurements of noise: CV, standard deviation (SD), and dispersion, α, given by

$$\alpha =\frac{\sigma^{2}-\mu }{\mu^{2}}$$

where σ is the SD and μ is the mean. These were calculated for each transcript over either the biological replicates or technical replicates to find the biological and technical noise, respectively. Similar results were obtained irrespective of the noise measure used (fig. S9).

#### GO analysis

GO analysis was done using the DAVID (*107*, *108*) annotation clustering tool. We used two normalization methods: normalization by DESeq2 and normalizing the raw read counts by the YFP transcript transcribed by a constitutively expressed promoter [P_R_ promoter (*92*); see “Strains and plasmids”; denoted “YFP” in the supplemented tables]. We consider a GO annotation cluster as significant if it has an enrichment score of >3 and false discovery rate (FDR) < 0.05 in both normalization methods. The two normalization methods gave similar annotation clusters, with a few differences for clusters with weak significance (see table S1 for a full list of detected clusters with their significance).

### Microcalorimetry measurements

Cultures were grown to either regulated or disrupted growth arrest as described in “Growth and growth-arrest conditions” or to exponential phase. One milliliter of samples from each 60-ml culture was transferred into 4-ml disposable glass vials, sealed, and introduced into the TAM IV microcalorimeter, at 32°C. The samples from the disrupted cultures were inserted into the calorimeter right after SHX was added to the medium. Regulated cultures were inserted into the calorimeter 4 hours after their OD saturated. The heat flow of the growth-arrested bacteria (Fig. 3E) was measured after 24 hours of growth arrest in the calorimeter. Colony-forming units (CFUs) were measured right before samples were inserted into the calorimeter and after 2 to 3 days in the calorimeter. To estimate the CFU after 24 hours, the initial and final CFUs were interpolated. The heat flow per cell was obtained by dividing the total heat flow by the CFU. Similarly, we measured the heat flow per cell in exponentially growing cultures by dividing the total heat flow by an estimate of the CFUs at that time. For this purpose, two technical replicates were loaded into the calorimeter at an OD of ~0.01. CFUs were measured before insertion into the calorimeter. One of the technical replicates was removed, and its CFU was measured after ~1 hour.

Heat flow measurements were performed using the Thermal Activity Monitor, TAM IV (TA Instruments). We used an assembly of six calorimeters (Minicalorimeter, 4 ml). The calorimeter’s design is described in (*109*). Experiments were performed in static ampoule mode.

### Survival during growth arrest

Three biological replicates were grown to either regulated or disrupted growth arrest as described in “Growth and growth-arrest conditions.” Samples were taken every 12 or 24 hours for CFU counting using serial dilution and plating. Results are presented in Fig. 3F.

### PI uptake assay

Cultures were grown in a 96-well plate to either regulated or disrupted growth arrest as described in “Growth and growth-arrest conditions.” After 24 hours in either regulated or disrupted growth arrest, PI (2.5 μg/ml) was added to half of the cultures. After 24 hours with PI (or additional 24 hours with no treatment for the control) a sample from each condition (regulated + PI, regulated, disrupted + PI, disrupted) was put on an agar pad with growth medium (without PI) and imaged under the microscope for 12 hours, where the temperature was controlled at 32°C. Cells that divided during this period were marked as alive. PI fluorescence intensity was measured for each cell before cells started to grow using image analysis. Only cells that were proven alive, i.e., divided during the time of measurement, are included in fig. S10. Control cultures with no PI showed no fluorescence.

### Measurements of survival fraction under drug treatments

Cultures were grown in a 96-well plate to either regulated or disrupted growth arrest. We conducted each experiment on three to four biological replicates. CFUs were counted by either plating a 100-μl sample at appropriate dilutions on fresh LB plates without antibiotics or counted using spotting serial dilutions. Spots with up to ~20 colonies were counted, where each spot contained 5 μl (x5 dilution was used between spots). CFUs were counted after a minimum of 48 hours of incubation at 37°C to ensure the detection of lagging cells.

The error bars in all survival fraction plots are the SEM of survival fraction for three to four biological replicates under each condition.

#### Survival fraction in ampicillin, SDS, vancomycin, and gentamicin during growth arrest

Drugs were added to the media after 24 hours of growth arrest to either regulated or disrupted cultures [ampicillin (50 μg/ml) (Fig. 1C), 1% SDS (Fig. 5I), gentamicin (5.2 μg/ml) (Fig. 5K), or vancomycin (1500 μg/ml, Fig. 5L)]. After 24 hours under treatment (or no treatment for controls), the CFUs were measured. The survival fraction was calculated as the ratio of CFU per milliliter after treatment to the CFU per milliliter of an untreated culture maintained in growth arrest for the same duration.

#### Survival fraction in ampicillin + nutrients

Regulated or disrupted cultures were diluted 1:100 into fresh medium with ampicillin (100 μg/ml), after CFU per milliliter was measured in each well (carryover SHX does not affect the growth, as shown in fig. S14). To stop the ampicillin exposure and prevent carryover, we added beta-lactamase (5 U/ml in 0.9% NaCl stock was diluted to a final concentration of 1 U/ml in the media) for 5 min before plating. The survival fraction was calculated as the ratio of CFU per milliliter after treatment to the CFU per milliliter before treatment (Figs. 1D and 5, D to F).

### SDS treatment during growth

Bacteria were grown overnight in LB, washed (5000*g*, for 3 min), and diluted 1:100 into M9 minimal media with 0.1% amino acids and kanamycin (15 μg/ml). Cells were grown at 37°C with shaking for 275 min before SDS was introduced. SDS was added to the culture from a stock of 10% in DDW to reach final concentrations of 1%. An equal volume of DDW was added to control cultures (fig. S13).

### Statistical tests

All statistical tests were performed using Python (scipy.stats package).

Mann-Whitney *U* test: We used the standard one-sided test.

Levene’s test: We used the standard test (where the mean is subtracted from each data point). Substituting the mean for the median or the trimmed mean did not change the test’s conclusions.

Permutation test: See “Determining the fraction of biologically variable genes” for details.

## Supplementary Materials

### The PDF file includes:

Supplementary Text Supplementary Methods Figs. S1 to S17 Table S5 Legends for movies S1 to S4 Legends for tables S1 to S4 References
