# Supplementary material for: Differentiation between regulated and disrupted growth arrests allows tailoring of effective treatments for antibiotic persistence
Source: Sci Adv. 2026 Jan 2;12(1):eadt6577. doi: 10.1126/sciadv.adt6577 (PMC12758538; doi:10.1126/sciadv.adt6577)
Supplement: Supplementary file 1 — Supplementary Text Supplementary Methods Figs. S1 to S17 Table S5 Legends for movies S1 to S4 Legends for tables S1 to S4 References [file sciadv.adt6577_sm.pdf]

Science Advances
AAAS

Supplementary Materials for

Differentiation between regulated and disrupted growth arrests allows tailoring of effective treatments for antibiotic persistence

Adi Rotem *et al.*

Corresponding author: Nathalie Q. Balaban, nathalie.balaban@mail.huji.ac.il

Sci. Adv. **12**, eadt6577 (2026)
DOI: 10.1126/sciadv.adt6577

The PDF file includes:

Supplementary Text
Supplementary Methods
Figs. S1 to S17
Table S5
Legends for movies S1 to S4
Legends for tables S1 to S4
References

Other Supplementary Material for this manuscript includes the following:

Movies S1 to S4
Tables S1 to S4

Supplementary Text

Random Graph Model of Cellular network Dynamics

Our goal is to illustrate the idea that the disrupted growth-arrest resembles a random exploration in the cell's configuration space (Fig. 2B), while regulated growth-arrest is a deep attractor of the dynamics (Fig. 2A). To this end, we present an illustrative model, that represents the dynamics of the cell's configuration space as if it were performing a random walk on a directional graph. In this graph, each node represents a certain configuration of all cellular compounds (for example mRNA, proteins levels etc.), in which certain pathways or genes are active, and the edges represent the transition probabilities between these configurations. Our goal is not to model the specific network of *E. coli,* but rather a random network of cellular compounds, representing abstract genes or pathways. The dynamics of a single cell during growth-arrest are represented by a trajectory in the cellular configurations graph.

We construct the graph randomly by drawing for each of the N nodes a random integer k from a Poisson distribution with mean λ. We then draw k incoming edges towards this node from other random nodes in the network and assign equal transition probabilities to each edge. Note that this procedure gives the directed analogue of the Erdős-Reyni graph, in the limit of $p \to 0, N \to \infty$ where pN is finite. While the volume of this configuration space is infinite, we expect the cell to visit only a finite number of cellular configurations. This justifies the representation of the system with a discrete graph that contains a finite (but large) number of nodes.

To solve the dynamics for the probability distribution of a population of cells we write the Master equation, where $\vec{p}_t$ represents the probability to find the cell in each cellular configuration at time t. The Master equation for the probability distribution of a random walker on the cellular configurations graph is given by:

$$\frac{d\vec{p}}{dt} = -\boldsymbol{L}\vec{\boldsymbol{p}} \qquad \text{Eq. 7}$$

Where the Laplacian matrix is defined as $L_{ij} = \delta_{ij} \sum_k A_{kj} - A_{ij}$, δ_{ij} is the Kronecker delta function, and $\boldsymbol{A}$ is the adjacency matrix of the cellular configurations graph, an $N \times N$ matrix where $A_{ij} = 1$ if a directed edge between node j and node i exists and $A_{ij} = 0$ otherwise. Notice that the definition of L implies that $\sum_j L_{ij} = 0$ and therefore the total probability is conserved. We numerically solve this set of ODEs and find the probability distribution of the random walk at a given time t. For the numerical solution, Eq. 7 is discretized as follows:

$$\vec{\boldsymbol{p}}_{t+1} = (\boldsymbol{I} + dt * \boldsymbol{L})\vec{\boldsymbol{p}}_t \qquad \text{Eq. 8}$$

where dt is the time interval between iterations and $\boldsymbol{I}$ is the identity matrix.

The random graph presented above represents the dynamics of a cell during a disrupted growth-arrest. As illustrated in fig. 2A, we model the regulated growth-arrest as an attractor within the same graph. To introduce an attractor to the graph, we manipulate the edges of one randomly chosen node. First, we remove the node's outgoing edges while leaving ingoing edges intact. We then repeat this procedure for all parent nodes up to a given depth d. This defines

the basin of attraction which on average is of size $\sum_{k=0}^{d-1} \lambda^k$. This results in a single node in the network that attracts adjacent states and is a fixed point of the dynamics (fig. S15). We model the two growth-arrests by selecting initial conditions that are either close to the attracting node (fig. S16A-C) or far away from it (fig. S16D-F). The regulated growth-arrest converges quickly into the attractor node, resulting in low variability (fig. S16C). In contrast, the disrupted growth-arrest performs a random diffusive process on the graph for a finite time, which leads to high variability between the final cellular configurations (fig. S16F).

We embed the cellular configurations graph onto an n dimensional vector space, that represents each of the relevant cellular components. In general, n could be a very large number that defines all the possible concentrations of mRNAs, proteins, metabolites, etc. To simplify our calculation, we choose an embedding dimension that is smaller than the number of nodes, and effectively resembles only the number of relevant components. The embedding is performed by the Kamada-Kawai algorithm (*110*), which is based on optimizing the deformations of a network of springs. This algorithm embeds the graph into a Euclidian space, where smaller distances represent stronger connectivity between the nodes.

Cell-to-cell variability of cellular components is predicted to be higher in the disrupted than in the regulated population

We now show that the cellular components in the disrupted growth-arrest are expected to have high cell-to-cell variability, while the regulated growth-arrest is expected to have low variability. For example, the induction of mCherry production is expected to be more variable in the disrupted bacteria. To find the variability of cellular components across single cells we use the embedded positions of cellular configurations, and the probabilities of each cellular configuration $\vec{\boldsymbol{p}}$. The variance across cells of a specific cellular component α is given by $Var(x^\alpha) = \sum_i p_i (x_i^\alpha - \langle x^\alpha \rangle)^2$, where x_i^α is the value of cellular component α in configuration i, and $\langle x^\alpha \rangle = \sum_i p_i x_i^\alpha$. Representative values of a randomly selected cellular component are presented in fig. S16C, F. As can be seen, the variance of the component values in the case of the modeled disrupted growth-arrest are significantly larger than the variance of values in the regulated growth-arrest (Fig. 2A) (see table S5 for a list of parameter values used). Our model's predictions align with the high cell-to-cell variability we observe in a single cellular component (mCherry production), in the disrupted but not in the regulated growth-arrest (Fig. 2E). Note that the promoter controlling the mCherry production is a generic constitutive promoter which is independent of additional regulation (*18*).

Sensitivity to initial conditions may result in higher variability between bulk biological replicates

Our model also predicts that the variability of cellular components in the disrupted growth-arrest should be observed even when averaged over many cells, as in the case of bulk culture measurements. This result may seem to go against the intuition that the average over millions of cells should not be variable. However, we show below that the structure of the disrupted cellular landscape may drive bulk cultures' biological variability. To model the noise between bulk measurements, we repeat the simulation many times, each time from a slightly

different initial condition, representing different biological replicates (fig. S17). Once we gain the average value of each cellular component, we calculate the std between biological replicates. Fig. 4A shows a bar plot of the std values of all d cellular components calculated over 40 biological replicates (see table S5 for a list of parameter values used). As can be seen, the variability of cellular components in the disrupted growth-arrest is larger than that of regulated growth-arrest. This aligns with our observations that gene expressions measured using RNA sequencing are variable between bulk biological replicates in disrupted (Fig. 4F) but not in regulated growth-arrest (Fig. 4E).

Gene expression in regulated and disrupted growth-arrest

When transitioning from a growth state to a growth-arrested state, whether disrupted or regulated, many genes exhibit significant changes. Among these, we were particularly interested in those that behave differently between regulated and disrupted growth-arrests. To this end, we devised a GO analysis scheme. First, we identify genes that are significantly changed in the transition from growth to growth-arrest for each of the growth-arrest conditions ($|\log_2 FC|$ of growth-arrest relative to growth is larger than 1, with p < 0.05, by DESeq2 (*65*)). We then narrow down our list of genes to those that are significantly different between the two growth-arrests ($\left|\log_2\left(\frac{\text{Disrupted}}{\text{regulated}}\right)\right| > 0.5$ with adjusted p-value < 0.05). These genes can be divided into four groups by their change relative to growing cells: 1. Increased in disrupted more than in regulated; 2. Increased in regulated more than in disrupted; 3. Decreased in disrupted more than in regulated; 4. Decreased in regulated more than in disrupted (See fig. S3A and table S1). We analyze each group using GO analysis (fig. S3B and table S1). To make our analysis more robust to the choice of normalization method, we repeat this process with a normalization by a constitutive gene (fig. S3C and D), and only consider GO terms that arise in both normalization methods. The resulting GO terms can reveal whether the regulated and disrupted growth-arrests differ in a biologically meaningful way.

We find that three groups of genes arise from this analysis: genes encoding for ribosomal proteins, flagella and chemotaxis (Fig. 3B-C, fig. S3B and D, and table S1). These two groups are found to significantly decrease in both regulated and disrupted growth-arrests, but much more so in regulated growth-arrest (fig. S3B and D). This finding is consistent between normalization methods (fig. S3B and D). This shows that cells in both growth-arrests respond to the stress by reducing their ribosomal proteins, flagellar and chemotaxis genes, as expected from growth-arrest responses (*2*, *4*, *25*, *40*, *43–48*, *53*). However, in regulated growth-arrest the response is more pronounced than in disrupted growth-arrest.

During the transition into stationary phase ribosomes are being inactivated and protected by a process called ribosome hibernation (*111*). We find that *rpmE*, and *ykgM* which are necessary for hibernation (*52*), as well as *ykgO* which is prevalent in stationary phase (*112*), and ribosome hibernation factors *raiA* and *rmf* (*51*) are among the few genes that are increased in regulated growth-arrest (Fig. 3C and table S3). These genes are unchanged, or even down

regulated, in disrupted growth-arrest, which is indicative that, despite their growth arrest, the ribosome hibernation process is inactive in disrupted bacteria.

A similar pattern is also found in genes encoding for chemotaxis and flagella. While most flagellar and chemotaxis genes are downregulated, as expected in stationary phase (*25*, *53*), the flagellar motor stator genes (*motAB*) (*113*, *114*), key chemotaxis components (*cheW*, *cheR*, *cheB* and *cheA*) (*115*), and methyl-accepting chemotaxis proteins (*tar*, *aer*, *tsr*, *trg* and *tap*) (*115*) are unchanged or even paradoxically upregulated in disrupted growth-arrest. This contradictory pattern supports the notion of a dysregulated system in the disrupted growth-arrest. In contrast, regulated growth-arrest displays coherent downregulation of flagella and chemotaxis functions, consistent with a controlled entry into growth-arrest.

Next, we asked whether the biologically variable genes (Fig. 4) are associated with particular biological processes. To address this, we analyzed the variable genes in the disrupted samples that were common across the two experiments, excluding the variable genes found in technical replicates or exponential-phase samples. Pathway enrichment analysis revealed that variable genes in the disrupted bacteria are significantly associated with bacterial chemotaxis and flagellar assembly (*cheB*, *cheY*, *cheZ*, *tar*, *flgN*, *ycgR*, *tap*, *flhB*, *flgL*, and *fliC*, hypergeometric test $p<0.0005$). Importantly, we observed almost no variable genes common to the two experiments in regulated growth-arrest, underscoring the transcriptional robustness of this state.

Supplementary Methods

Determining the fraction of biologically variable genes

For each condition we calculated the observed number of genes considered biologically variable (for which the biological noise was 4 times greater than the technical noise, where the noise was calculated by either the CV, the STD or the dispersion). We then shuffled the group indexes a 1000 times for each gene between biological and technical samples, and calculated the number of genes biologically variable for each permutation. The p-value was calculated as the fraction of permutations with the observed number of biologically variable genes or larger. In all tests results $p < 0.001$.

Microfluidics - Fitting the single-cell intensity curves

The single-cell fluorescence intensity curves of the measurements presented in Fig. 2, D to H are fitted to a function with a sigmoidal derivative (see fig. S1):

$$\frac{dp}{dt} = a\left(\tanh\left(\gamma(t - t_0)\right) + 1\right) \qquad \text{Eq. 1}$$

Where p is the measured fluorescence intensity per pixel, and a, γ, t_0 are fitting parameters. This assumption (and $\lim_{t\to-\infty} p(t) = 0$) implies that the fluorescence intensity goes as:

$$p(t) = a \cdot \frac{1}{\gamma}\left(\log\left(\cosh\left(\gamma(t - t_0)\right)\right) + \gamma(t - t_0) + \log 2\right) \qquad \text{Eq. 2}$$

We use least square fitting to find the fit parameters a, t_0 and γ for each measured curve.

RNA-seq experiments repeat

The RNA-seq experiment was repeated twice, with slight variations as described below. In the first experiment (available at ArrayExpress, accession E-MTAB-14335) three biological replicates were grown to either regulated or disrupted growth-arrest, where three technical replicates were collected from an additional biological replicate (this was done in both regulated and disrupted growth-arrest). The data from this experiment in shown in fig. S7. In the second experiment (available at ArrayExpress, accession E-MTAB-14332) three biological replicates were grown to either regulated or disrupted growth-arrest, where three technical replicates were taken from biological replicate 1. The biological replicates used in this experiment are distinct from the ones used in the first experiment. In this repetition samples were also collected at the growth phase, right before SHX was added to the disrupted samples. The results of this experiment are used in the analysis presented in Fig. 3A-C and in Fig. 4, B to F.

Absolute gene expression normalization using ERCC spike-ins

ERCCs were added to all samples at fixed amounts per total RNA, after RNA extraction and before rRNA depletion. For each sample we calculated the RPK of raw reads of each ERCC transcript. The number of molecules in each sample for each ERCC transcript are known from the ERCC datasheet provided by ThermoFisher. We then use least square fitting to fit:

$$\log(\mathrm{RPK}) = m \cdot \log(\text{molecules per sample}) + c$$

The fit was performed for all detected spike-ins with molecules per sample > 0.01, for each sample separately. All fits were with $R^2 > 0.96$. An example of a plot of the measured RPK as a function of the number of molecules in the sample is presented in fig. S2. We then calculated the RPK of all transcripts, and converted the RPK for each sample to molecules per sample using:

$$\text{molecules per sample} = e^{-\frac{c}{m}} \cdot (\mathrm{RPK})^{\frac{1}{m}} \qquad \text{Eq. 3}$$

Next, we estimated the number of cells in each sample, to get an estimate of the number of molecules per cell. This was done by measuring the CFUs in the original culture and multiplying it by the volume collected from the culture. By knowing the volume to which ERCC were added to, we then get an estimate of the molecules per cell:

$$\text{molecules per cell} = \frac{\text{molecules per sample}}{\text{CFU per sample}} \qquad \text{Eq. 4}$$

Finally, for a more robust sample size estimation, we used a total counts correction similar to the one used by DESeq2 size factor correction. We assume that different samples in the same condition have similar total amounts of molecules per cell, and calculate a size factor for each sample according to this assumption:

$$S_i = \frac{T_i}{\frac{1}{N}\sum_i T_i} \qquad \text{Eq. 5}$$

Where S_i is the size factor of sample i , and T_i is the total amount of molecules per sample in sample i, N is the number of samples, and the sum is over all samples of the same condition. The molecules per cell of each transcript in each sample are then divided by the size factor of that sample. Non-coding transcripts were filtered out.

The code used to normalize the data is available at the Zenodo repository. The data normalized by this method (table S2) is used in fig. S5, E to F. For Fig. 3A we used the spike-in normalization presented here without the last step of total count correction.

Supplementary Figures

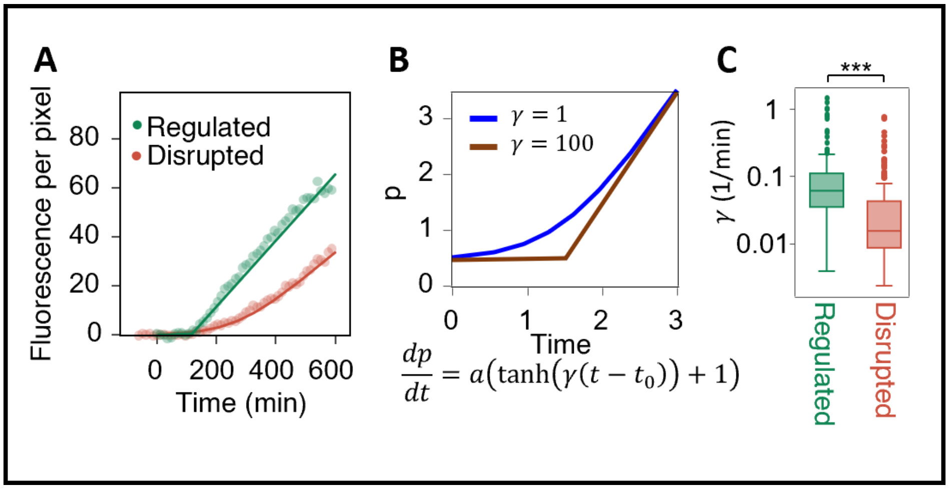

fig. S1. Peak protein production rate is reached quickly in regulated growth-arrest, and lags in disrupted growth-arrest. (**A**) Typical examples of single-cell fluorescence induction measurements of disrupted and regulated bacteria, with a fit to the function described in (B) (Eq. 1, solid lines). In disrupted bacteria, the maximal slope is reached only several hours after induction. (**B**) Two schematic examples of induction of protein production, with different values of γ. (**C**) Measured distribution of γ for regulated and disrupted bacteria. γ is significantly larger for regulated bacteria (Mann Whitney U test, $U = 9172$, $p < 0.005$).

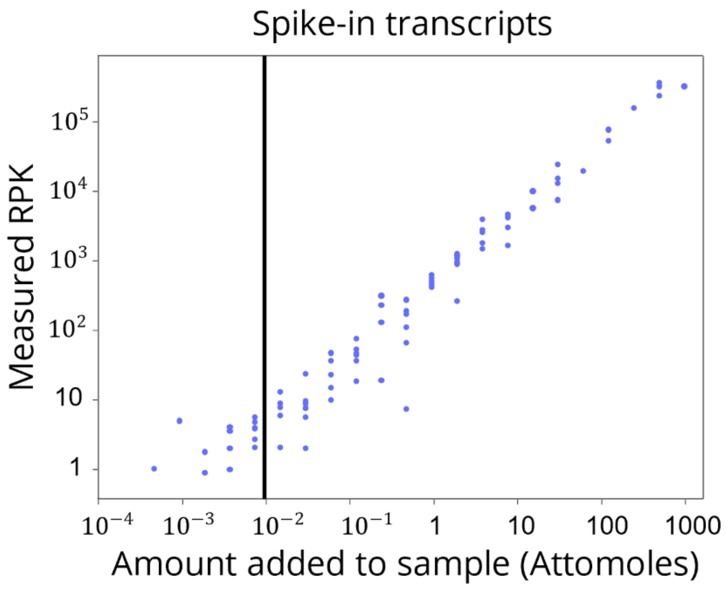

fig. S2. Spike-ins allow estimating the detection limit in bulk RNA samples. ERCC spike-ins consist of 92 transcripts of varying concentration (*41*). These spike-ins were added in known quantities to each sample before sequencing. The plot shows the measured RPK of each spike-in as a function of its known concentration in the sample, for a specific example of one of the regulated samples. Spike-ins with concentrations exceeding 0.01 attomoles per sample were consistently detected in all samples (black line), and this value was set as the detection limit.

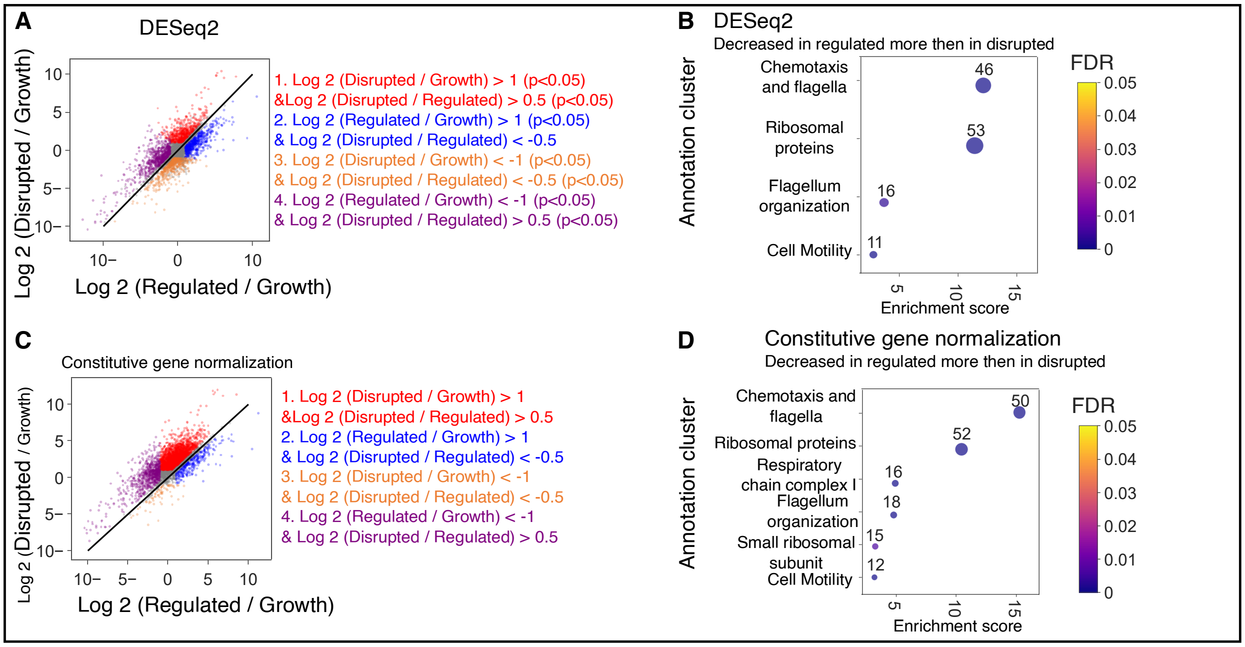

fig. S3. GO analysis: Ribosomal protein, chemotaxis and flagellar genes are decreased (compared to exponential growth) in the regulated more than in the disrupted growth-arrest. (A) Genes were divided into four groups: 1. Increased in disrupted more than in regulated; 2. Increased in regulated more than in disrupted; 3. Decreased in disrupted more than in regulated; 4. Decreased in regulated more than in disrupted (DESeq2 normalization; only genes with significant fold changes of $p < 0.05$ were considered). For each of the four groups, we ran GO analysis annotation clustering. **(B)** Annotation clusters found for the group of genes that were decreased in regulated more than in disrupted growth-arrest. The plot shows the annotation cluster names, false detection rate (FDR) for each cluster and the enrichment score. Only significant clusters are shown (enrichment score > 3, FDR < 0.05). **(C)** Same as in (A), but for gene expression where each gene was normalized by the value of a constitutively expressed gene. **(D)** Same as in (B), but for the constitutive gene normalization. Note that only group 4 is shown since it was the only group of genes that produced annotation clusters that were significant in both normalizations. The full list of genes in each annotation cluster in (B, D) and the gene groups in (A, C) is provided in table S1.

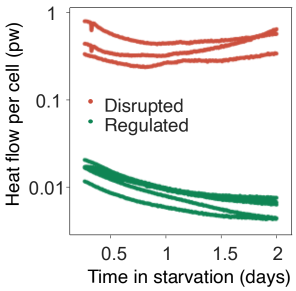

fig. S4: Heat production in disrupted growth-arrest remains larger than in regulated growth-arrest for days. Heat production per cell, as measured by total heat measurement using microcalorimetry and division by the number of CFUs (see Methods). Cultures were grown to either regulated or disrupted growth-arrest, and subsequently placed in the microcalorimeter. Each trace represents a different biological replicate.

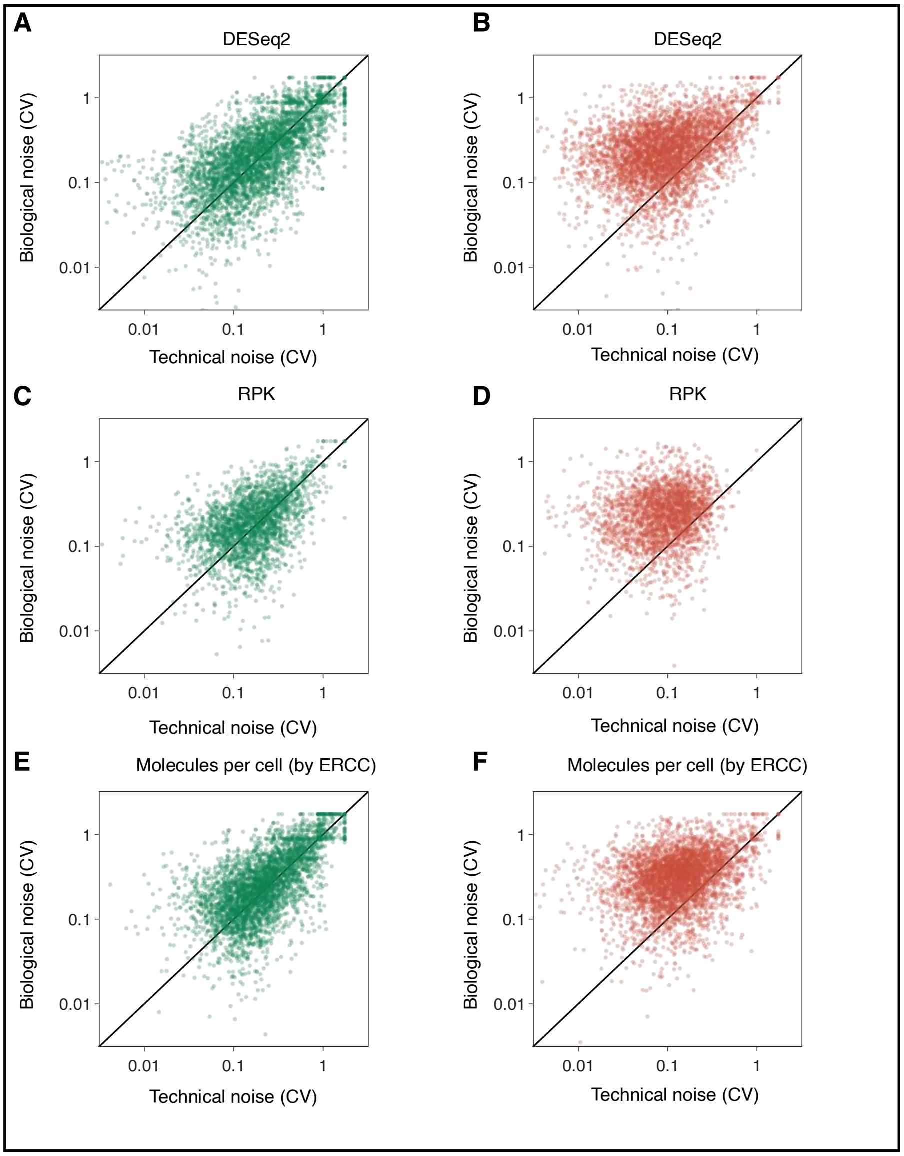

fig. S5. Noise is increased in disrupted versus regulated growth-arrest, regardless of the normalization method. The CV of biological replicates noise as a function of the CV of technical noise for all protein coding genes in (**A, C, E**) regulated (green) or (**B, D, F**) disrupted (red) growth-arrest for the same data presented in Fig. 4. Results are shown in (A, B) DESeq2 normalization, (C, D) Reads per kilo base pair (RPK) and (E, F) Molecules per cell, as calculated using ERCC spike-ins normalization. The diagonal line is the identity line. In regulated growth-arrest in all normalization no more than 7% of genes have biological noise that exceeds the technical noise (higher than four-fold biological to technical CV, p-values of group permutation test < 0.001), for both the experiment presented in Fig. 4 and its repetition presented in fig. S7. In disrupted growth-arrest at least 30% of genes are noisy, where the exact percent of noisy genes is

normalization and experiment dependent (Same significance test. DESeq2: 30-40%, RPK: 31-32%, molecules per cell: 30-34%).

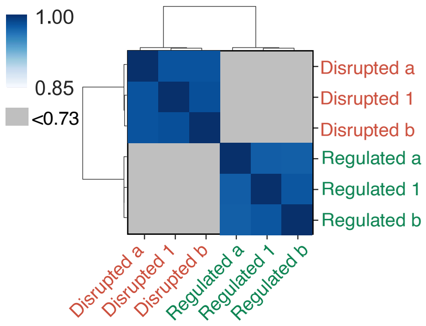

fig. S6. Technical errors are low in both regulated and disrupted growth-arrests. Spearman correlations between technical replicates of all protein coding genes with more than 50 aligned raw reads.

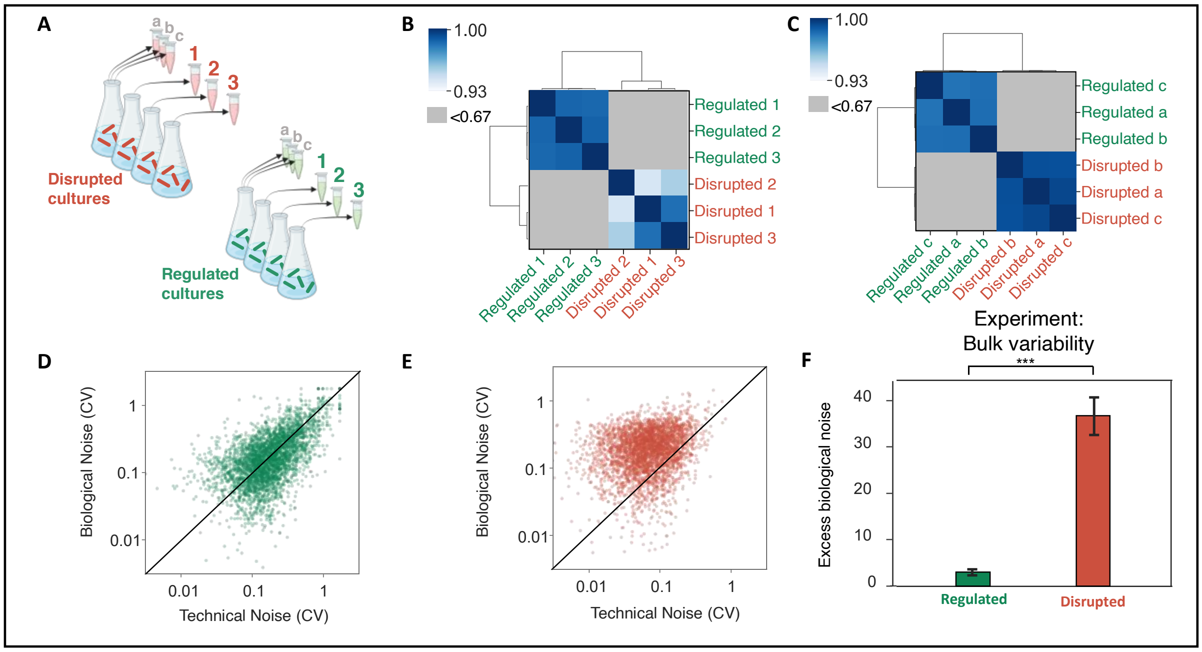

fig. S7. Repeating the full gene expression noise experiment reproduces the results of increased noise in disrupted growth-arrest. (**A**) Four biological replicates were grown to either regulated or disrupted growth-arrest and measured by RNA sequencing ("biological replicates"). Three technical samples from one of the biological replicates were also collected ("technical replicates"). (**B**) Spearman correlation between biological replicates of all protein coding genes with more than 50 aligned raw reads. Biological replicates of disrupted growth-arrest are significantly less correlated (Mann-Whitney $p = 0.05$) (**C**) Same as in (B), but for the technical replicates. (**D** and **E**) The biological noise (CV of expression over biological samples) versus the technical noise (CV of expression over technical samples) of each gene in either (D) regulated or (E) disrupted samples. The black line is the identity curve. In the regulated cultures 4% of genes had higher than 4-fold biological to technical CV (permutation test $p < 0.001$) whereas in disrupted cultures 31% of genes were noisy (same significance test). (**F**) The excess biological noise of genes in the disrupted and regulated growth-arrests, as defined by (biological variance – technical variance)/mean expression. (***: Mann Whitney $U = 2336687$, $p < 0.0001$). In (B-F) we used down-sampling normalization (the conclusions are the same for different choices of normalization fig. S5).

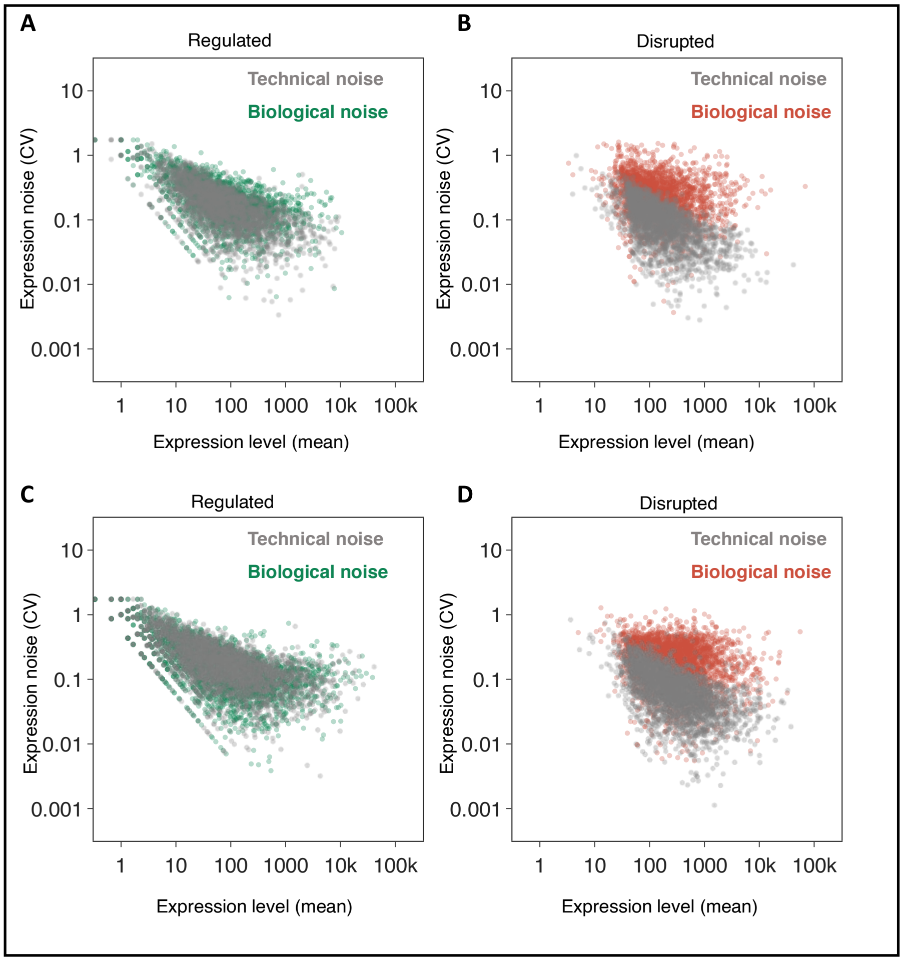

fig. S8. The biological noise in the disrupted growth-arrest is apparent at all expression levels. CV vs. mean of all genes for either (**A**, **C**) regulated or (**B**, **D**) disrupted growth-arrest, where the CV and mean are either calculated over biological (green for regulated and red for disrupted) or technical (grey). (A, B) show the results for the experiment described in Fig. 4, and (C, D) show the same plots for the repetition of this experiment as described in fig. S7. The resulted are presented in raw read units, down-sampled to the lowest sampling depth.

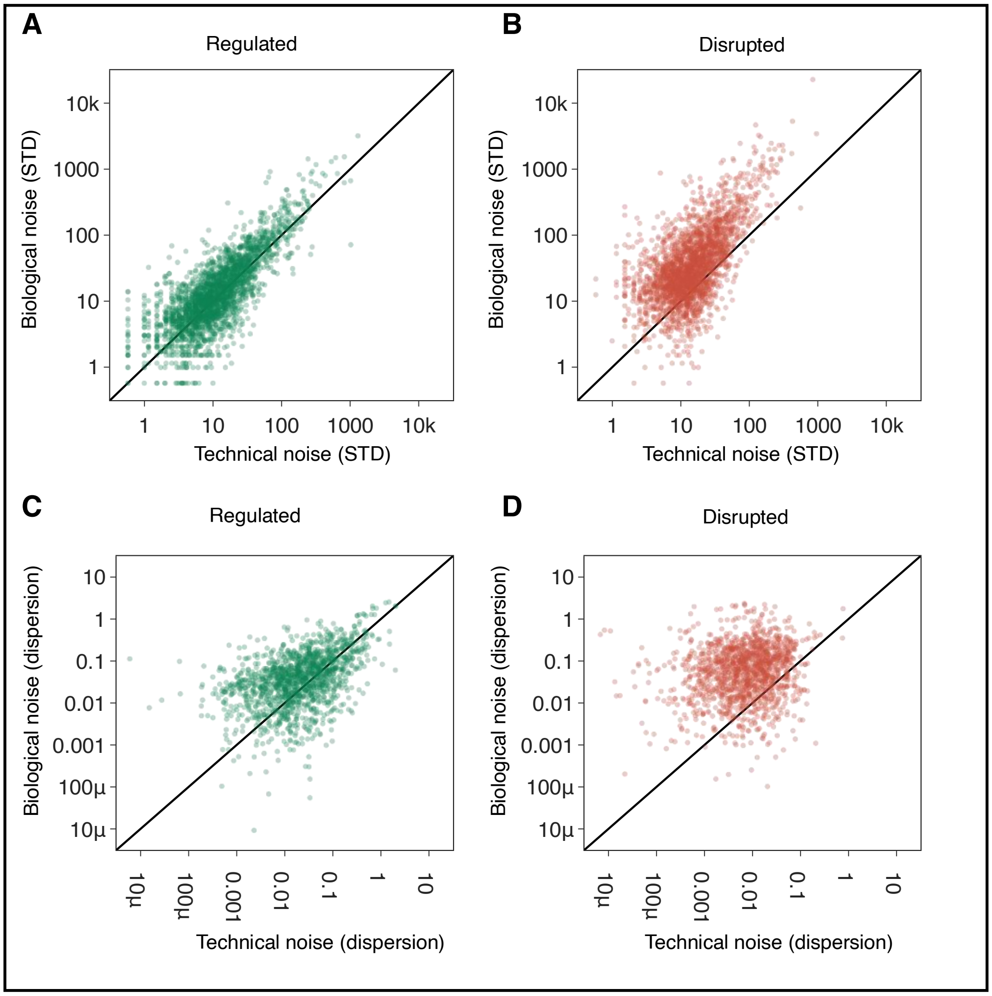

fig. S9. The biological noise in the disrupted growth-arrest is apparent regardless of the noise metric. Biological vs. technical noise of all protein coding genes in either regulated (**A**, **C**) and disrupted (**B**, **D**) arrest cultures for the data presented in Fig. 4. Both noise metrics, STD (A, B) and dispersion (C, D) reproduce the increased variability observed in the disrupted growth-arrest. The same plots for the repetition of the experiment (fig. S7) showed similar results. For all noise measurements presented in this figure, and for both repetitions of the experiment, the regulated growth-arrest had significantly less noisy genes than the disrupted growth-arrest (group permutation test, $p < 0.001$, see supplementary methods).

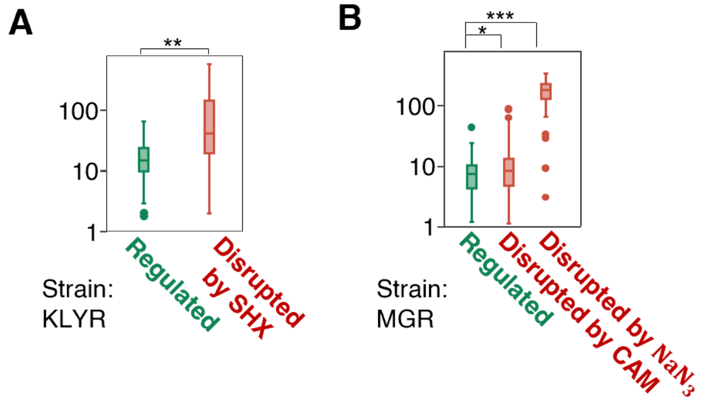

fig. S10. The membranes of disrupted bacteria are more permeable than the membranes of regulated bacteria. (A) Live *E. coli* KLYR regulated growth-arrested bacteria show a low PI fluorescence whereas live bacteria disrupted by SHX show higher PI signal, as measured by fluorescence microscopy. Cells were in growth-arrest for 24 hours, and treated with PI for additional 24 hours, before being transferred into fresh media (without PI) under the microscope. The fluorescence intensity is shown for cells that are alive, defined by a division observed during the next 10 hours. (B) same as in (A), but for *E. coli* MGR that were grown to regulated growth-arrest or disrupted by either CAM or NaN_3 (cells were in growth-arrest for 24 hours). Horizontal line: median; box: 25% and 75% quartiles; whiskers: 1.5 interquartile range (IQR); Outliers: > 1.5 IQR. (*: Mann Whitney $p < 0.05$, **: $p < 0.005$, ***: $p < 0.0005$, $n > 50$ in all conditions).

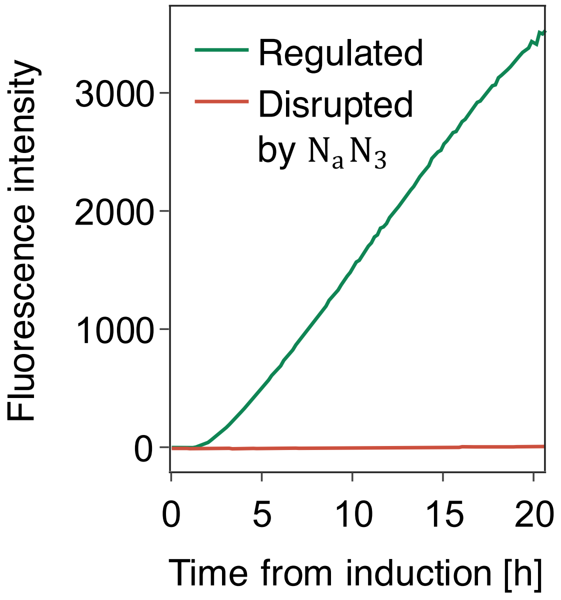

fig. S11. Protein induction is inhibited in cells treated with NaN_3. *E.coli* MG1655/pZA21RmCherry were grown to regulated growth-arrest (green) or treated with NaN_3 (10.5 mM, red). After 29 hours in growth-arrest (*t*=0), protein production was induced by aTc (the strain contains an inducible fluorescent protein on a plasmid, described in Fig. 1F inset). Regulated growth-arrested cells produce protein, but cells disrupted with NaN_3 do not.

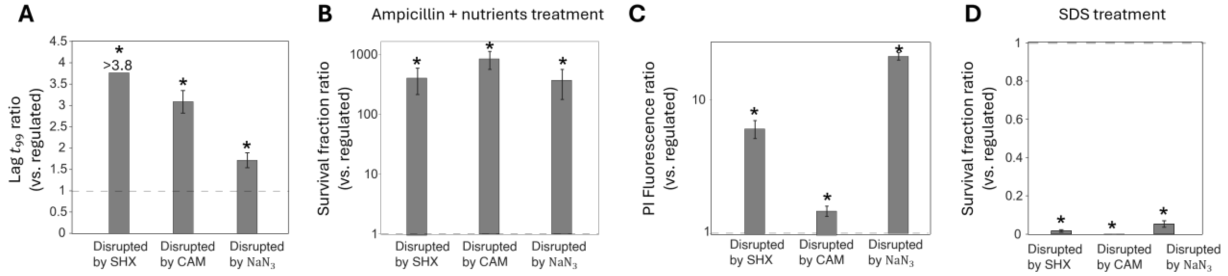

fig. S12. Disrupted growth-arrests exhibit broader recovery times, antibiotic tolerance upon regrowth, increased membrane permeability, and enhanced hypersensitivity relative to regulated growth-arrests. (**A**) Ratio of t_{99} (time at which 99% of cells resume growth) of disrupted to regulated growth-arrests. t_{99} values were extracted from lag-time distributions (Fig. 5A to C). Values exceeding the measurement window are indicated with a ">" above the bar. All ratios are significantly greater than 1 (Mann–Whitney, $p<0.05$), as a result of the larger t_{99} of disrupted cells. Error bars represent SEM from three or more biological replicates. (**B**) Ratio of survival fraction in fresh medium with ampicillin (100 µg/ml, 6 hours), calculated from Fig. 5D to F. All ratios are significantly larger than 1 (Mann–Whitney, $p<0.05$), showing that disrupted cells are orders of magnitude less susceptible to this treatment than regulated cells. (**C**) Ratio of mean PI fluorescence per pixel (disrupted/regulated) from PI-treated cells, calculated from the data in fig. S10. All ratios are significantly greater than 1 (permutation test using the disrupted/regulated mean ratio as the test statistic, $p<0.05$), indicating increased PI permeability in disrupted conditions. Error bars were obtained by error propagation of SEM across individual cells. (**D**) Ratio of survival fractions in 1% SDS (disrupted/regulated), calculated from the data in Fig. 5I. All ratios are significantly less than 1 (Mann–Whitney, $p<0.05$), showing that disrupted cells are more sensitive than regulated cells. In all panels, the dashed line denotes a ratio of 1.

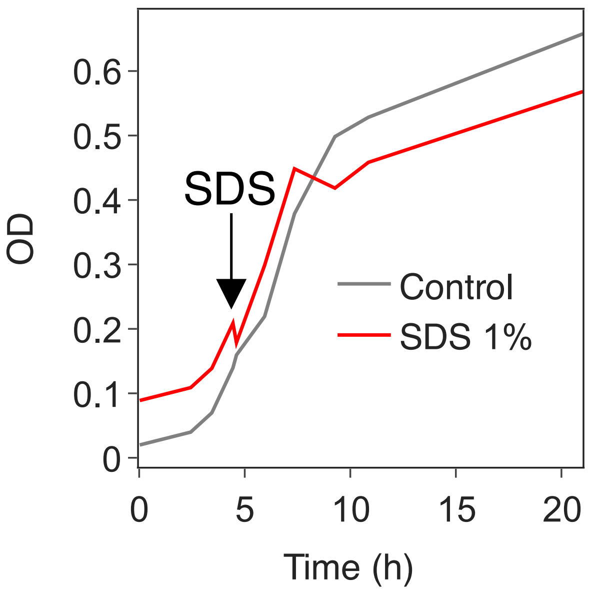

fig. S13. The MIC of SDS for *E. coli* is above 1% SDS. *E. coli* MG1655intRmCH were grown in minimal media which was supplemented after 5h (arrow) with 1% SDS or a control (DDW). Cells continue to grow in the presence of 1% SDS. However, the same concentration results in the death of disrupted bacteria (Fig. 5I)

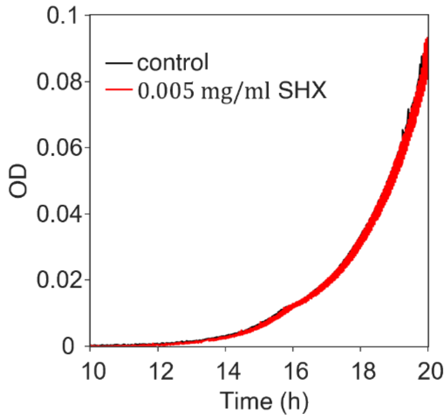

fig. S14. Cell growth is unaffected by 0.005mg/ml of SHX. *E. coli* KLYR were grown in minimal media with 0.1% amino-acids, with (red) or without (black) 0.005 mg/ml of SHX. The growth is unaffected by this concentration of SHX. The figure shows three overlayed biological replicates.

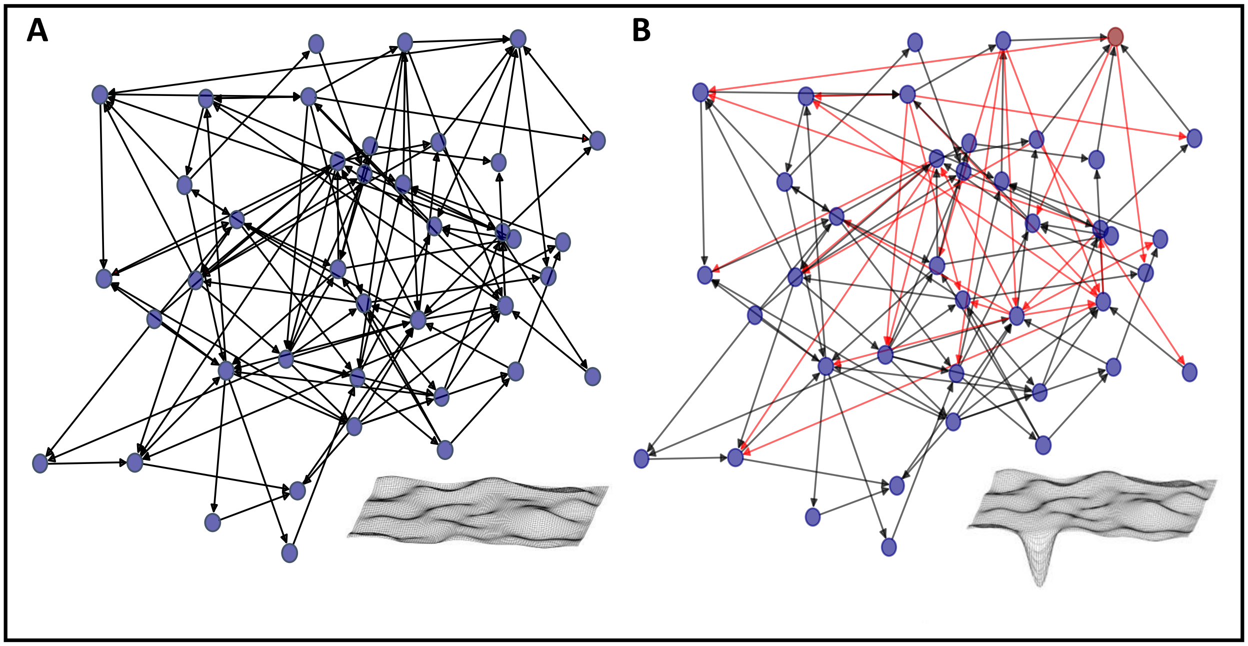

fig. S15. Visualization of the graph representing growth-arrest dynamics. (**A**) a random directed graph is generated with 40 nodes, λ=3 average node degree, visualized using an embedding onto the 2-dimensional plane. Inset: a schematic representation of the random landscape. (**B**) to add an attractor to the graph, a random node is selected (marked in red) and the outgoing edges of this node and of its successors up to a depth d=2 are removed (marked in red). Inset: a schematic representation of the random landscape with the attractor.

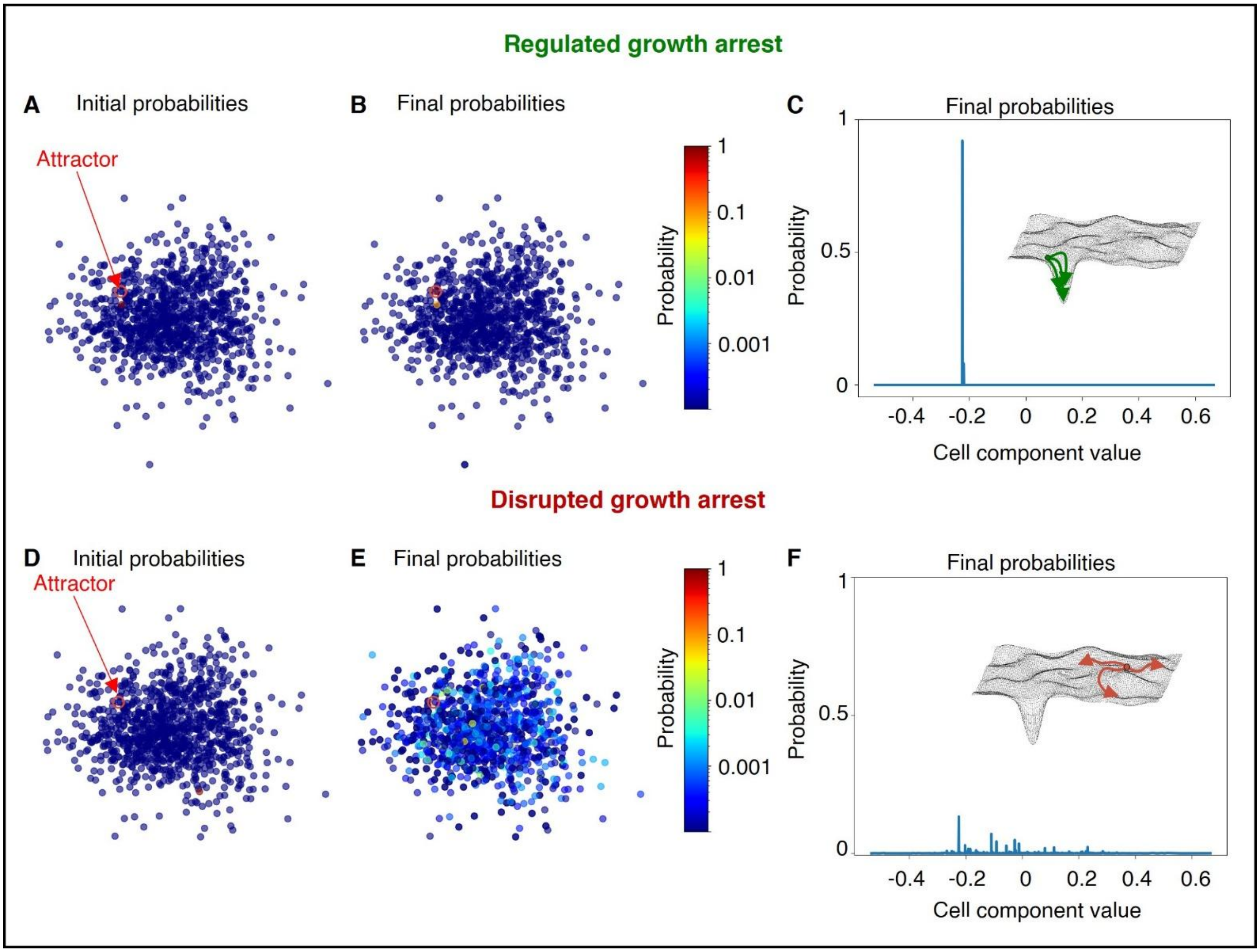

fig. S16. Dynamics of the diffusion process for regulated and disrupted growth-arrests. A network consisting of $\boldsymbol{N = 1000}$, $\boldsymbol{\lambda = 3}$, with an attractor of depth $d = 3$ is generated. (**A-B**) Visualization of the network using a 2-dimensional embedding (the edges are hidden to improve the clarity of the graph). The probability vector is used to color the nodes. (A) Shows the initial state of the system where the system is initialized close to the attractor, representing the regulated growth-arrest. (B) The final probabilities after 250 time steps. (**C**) The endpoint of the probability vector is plotted against a randomly chosen dimension of the embedding (a single cell component value). Inset: schematic representation of the dynamics showing that when the system is initialized close to the attractor the probability to find the system inside the attractor is large. (**D, E** and **F**) Same as in (A, B and C) respectively, but when the simulation is initialized far from the attractor, representing the disrupted growth-arrest. The inset in (F) shows that when the system is initialized far from the attractor, it can be at any state at the final configuration.

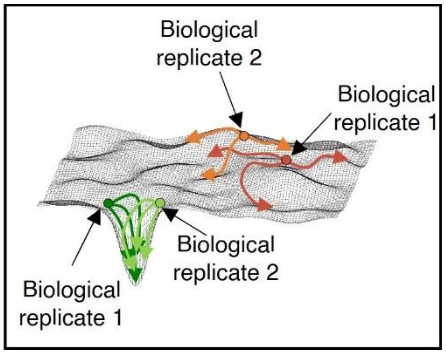

fig. S17. Schematic representation of different biological replicates in the model. The system is initialized at different initial conditions, representing different biological replicates. Different biological replicates starting near the attractor are expected to have lower variability than different biological replicates initiated far from the attractor.

	Description	Single cell Variability	Bulk Variability
$\boldsymbol{N}$	Number of nodes	1000	1000
$\boldsymbol{\lambda}$	Mean incoming edges per node	4	4
$\boldsymbol{d}$	Attractor depth	4	4
$\boldsymbol{\tau}$	Time steps	500	500
$\boldsymbol{dt}$	Time interval	0.01	0.01

Table S5. A list of parameters used in the simulations.

Movie S1. (separate file)

Monitoring fluorescent protein induction of regulated growth-arrested bacteria at the single-cell level. Microscopy timelapse imaging of bacteria in a microfluidics device under growth conditions (t = 0 −144 min), followed by regulated growth-arrest. At t = 396 min, the inducer was added (aTc) triggering mCherry production during the regulated growth-arrest. Images of phase contrast (grey), constitutive YFP expression (green), and mCherry induction (red) are overlaid. Most bacteria start producing mCherry at the same time and at a constant rate upon induction.

Movie S2. (separate file)

Monitoring fluorescent protein induction of disrupted growth-arrested bacteria at the single-cell level. Microscopy timelapse imaging of bacteria in a microfluidics device under growth conditions (t = 0 −108 min), followed by disrupted growth-arrest. At t = 360 min, the inducer was added (aTc) triggering mCherry production during the disrupted growth-arrest. Images of phase contrast (grey), constitutive YFP expression (green), and mCherry induction (red) are overlaid. Some bacteria start producing mCherry immediately upon induction, while others require more time.

Movie S3. (separate file)

Regulated growth-arrested cells have intact membranes. Typical microscopy timelapse imaging of PI stained regulated bacteria, in phase contrast (grey) overlaid with PI staining (red). At time t = 0 PI treated regulated bacteria were taken from a growth-arrested batch culture to an agar pad with fresh nutrients. Cells are not stained by PI, showing that cells in regulated growth-arrest have intact membranes. Scale bar: $5\mu m$.

Movie S4. (separate file)

Disrupted growth-arrested cells have permeable membranes. Typical microscopy timelapse imaging of PI stained disrupted bacteria, in phase contrast (grey) overlaid with PI staining (red). At time t=0 PI treated disrupted bacteria were taken from a growth-arrested batch culture to an agar pad with fresh nutrients. Some cells are stained by PI (red overlay) but start to grow nevertheless. This shows that cells in the disrupted growth-arrest have permeable membranes but are still alive and are able to regrow. Scale bar: $5\mu m$.

Table S1. (separate file)

GO analysis. A list of genes used for GO analysis and GO clusters found for these genes, as described in fig. S3.

Table S2. (separate file)

Gene expression in molecules per cell. Gene expression of protein coding transcripts in molecules per cell, as normalized by ERCC spike-ins (see materials and methods, under "Absolute gene expression normalization using ERCC spike-ins"). Each sheet shows a different repetition of the experiment ("experiment1" and "experiment2", see materials and methods).

Table S3. (separate file)

Differential gene expression calculated using DESeq2. The base mean, log2 fold change and adjusted p-values for regulated or disrupted growth-arrest as treatment, and exponential growth as a control.

Table S4. (separate file)

Gene expression in raw reads. Gene expression of protein coding transcripts in down-sampled raw reads (see materials and methods, under "Normalization methods for gene expression relative to total sample expression"). Each sheet shows a different repetition of the experiment ("experiment1" and "experiment2", see materials and methods).

REFERENCES

1. C. N. Peterson, M. J. Mandel, T. J. Silhavy, *Escherichia coli* starvation diets: Essential nutrients weigh in distinctly. *J. Bacteriol.* **187**, 7549–7553 (2005).

2. R. Hengge, Stationary-phase gene regulation in *Escherichia coli*. *EcoSal Plus* **4**, 10.1128/ecosalplus.5.6.3 (2011).

3. T. Ferenci, Hungry bacteria—Definition and properties of a nutritional state. *Environ. Microbiol.* **3**, 605–611 (2001).

4. K. Potrykus, M. Cashel, (p)ppGpp: Still magical? *Annu. Rev. Microbiol.* **62**, 35–51 (2008).

5. R. Kolter, D. A. Siegele, A. Tormo, The stationary phase of the bacterial life cycle. *Annu. Rev. Microbiol.* **47**, 855–874 (1993).

6. T. Nyström, Stationary-phase physiology. *Annu. Rev. Microbiol.* **58**, 161–181 (2004).

7. D. E. Jenkins, J. E. Schultz, A. Matin, Starvation-induced cross protection against heat or H2O2 challenge in Escherichia coli. *J. Bacteriol.* **170**, 3910–3914 (1988).

8. J. Jaishankar, P. Srivastava, Molecular basis of stationary phase survival and applications. *Front. Microbiol.* **8**, 2000 (2017).

9. R. Lange, R. Hengge-Aronis, Identification of a central regulator of stationary-phase gene expression in *Escherichia coli*. *Mol. Microbiol.* **5**, 49–59 (1991).

10. K. Lewis, Persister cells, dormancy and infectious disease. *Nat. Rev. Microbiol.* **5**, 48–56 (2007).

11. S. Kjelleberg, N. Albertson, K. Flärdh, L. Holmquist, Å. Jouper-Jaan, R. Marouga, J. Östling, B. Svenblad, D. Weichart, How do non-differentiating bacteria adapt to starvation? *Antonie Van Leeuwenhoek* **63**, 333–341 (1993).

12. R. Hengge-Aronis, R. Lange, N. Henneberg, D. Fischer, Osmotic regulation of rpoS-dependent genes in Escherichia coli. *J. Bacteriol.* **175**, 259–265 (1993).

13. T. Nyström, N. Gustavsson, Maintenance energy requirement: What is required for stasis survival of *Escherichia coli*? *Biochim. Biophys. Acta* **1365**, 225–231 (1998).

14. M. M. Zambrano, R. Kolter, GASPing for life in stationary phase. *Cell* **86**, 181–184 (1996).

15. M. M. Zambrano, D. A. Siegele, M. Almirón, A. Tormo, R. Kolter, Microbial competition: *Escherichia coli* mutants that take over stationary phase cultures. *Science* **259**, 1757–1760 (1993).

16. S. Avrani, E. Bolotin, S. Katz, R. Hershberg, Rapid genetic adaptation during the first four months of survival under resource exhaustion. *Mol. Biol. Evol.* **34**, 1758–1769 (2017).

17. S. E. Finkel, Long-term survival during stationary phase: Evolution and the GASP phenotype. *Nat. Rev. Microbiol.* **4**, 113–120 (2006).

18. S. Berthoumieux, H. De Jong, G. Baptist, C. Pinel, C. Ranquet, D. Ropers, J. Geiselmann, Shared control of gene expression in bacteria by transcription factors and global physiology of the cell. *Mol. Syst. Biol.* **9**, 634 (2013).

19. K. Lewis, *Persister Cells and Infectious Disease* (Springer Nature, 2019).

20. R. A. Fisher, B. Gollan, S. Helaine, Persistent bacterial infections and persister cells. *Nat. Rev. Microbiol.* **15**, 453–464 (2017).

21. N. Q. Balaban, J. Merrin, R. Chait, L. Kowalik, S. Leibler, Bacterial persistence as a phenotypic switch. *Science* **305**, 1622–1625 (2004).

22. V. Chubukov, U. Sauer, Environmental dependence of stationary-phase metabolism in *Bacillus subtili*s and *Escherichia coli*. *Appl. Environ. Microbiol.* **80**, 2901–2909 (2014).

23. A. J. Saldanha, M. J. Brauer, D. Botstein, Nutritional homeostasis in batch and steady-state culture of yeast. *Mol. Biol. Cell* **15**, 4089–4104 (2004).

24. S. M. Amato, M. A. Orman, M. P. Brynildsen, Metabolic control of persister formation in *Escherichia coli*. *Mol. Cell* **50**, 475–487 (2013).

25. A. Battesti, N. Majdalani, S. Gottesman, The RpoS-mediated general stress response in Escherichia coli. *Annu. Rev. Microbiol.* **65**, 189–213 (2011).

26. G. Storz, R. Hengge, Eds., *Bacterial Stress Responses* (ASM Press, ed. 2, 2010).

27. R. Hengge-Aronis, Signal transduction and regulatory mechanisms involved in control of the σ^S (RpoS) subunit of RNA polymerase. *Microbiol. Mol. Biol. Rev.* **66**, 373–395 (2002).

28. S. E. Irving, N. R. Choudhury, R. M. Corrigan, The stringent response and physiological roles of (pp)pGpp in bacteria. *Nat. Rev. Microbiol.* **19**, 256–271 (2021).

29. L. U. Magnusson, A. Farewell, T. Nyström, ppGpp: A global regulator in *Escherichia coli*. *Trends Microbiol.* **13**, 236–242 (2005).

30. J. M. Navarro Llorens, A. Tormo, E. Martínez-García, Stationary phase in gram-negative bacteria. *FEMS Microbiol. Rev.* **34**, 476–495 (2010).

31. Y. Kaplan, S. Reich, E. Oster, S. Maoz, I. Levin-Reisman, I. Ronin, O. Gefen, O. Agam, N. Q. Balaban, Observation of universal ageing dynamics in antibiotic persistence. *Nature* **600**, 290–294 (2021).

32. J. L. Radzikowski, H. Schramke, M. Heinemann, Bacterial persistence from a system-level perspective. *Curr. Opin. Biotechnol.* **46**, 98–105 (2017).

33. S. Reich, S. Maoz, Y. Kaplan, H. Rappeport, N. Q. Balaban, O. Agam, Slow relaxation and aging in the model of randomly connected cycles network. *Phys. Rev. Res.* **4**, 033127 (2022).

34. O. Gefen, O. Fridman, I. Ronin, N. Q. Balaban, Direct observation of single stationary-phase bacteria reveals a surprisingly long period of constant protein production activity. *Proc. Natl. Acad. Sci. U.S.A.* **111**, 556–561 (2014).

35. C. H. Waddington, *The Strategy of the Genes* (Routledge, 2014).

36. P. Wang, L. Robert, J. Pelletier, W. L. Dang, F. Taddei, A. Wright, S. Jun, Robust growth of *Escherichia coli*. *Curr. Biol.* **20**, 1099–1103 (2010).

37. T. M. Norman, N. D. Lord, J. Paulsson, R. Losick, Memory and modularity in cell-fate decision making. *Nature* **503**, 481–486 (2013).

38. B. Okumus, C. J. Baker, J. C. Arias-Castro, G. C. Lai, E. Leoncini, S. Bakshi, S. Luro, D. Landgraf, J. Paulsson, Single-cell microscopy of suspension cultures using a microfluidics-assisted cell screening platform. *Nat. Protoc.* **13**, 170–194 (2018).

39. S. Bakshi, E. Leoncini, C. Baker, S. J. Cañas-Duarte, B. Okumus, J. Paulsson, Tracking bacterial lineages in complex and dynamic environments with applications for growth control and persistence. *Nat. Microbiol.* **6**, 783–791 (2021).

40. B. Gummesson, S. A. Shah, A. S. Borum, M. Fessler, N. Mitarai, M. A. Sørensen, S. L. Svenningsen, Valine-induced isoleucine starvation in *Escherichia coli* K-12 studied by spike-in normalized RNA sequencing. *Front. Genet.* **11**, 144 (2020).

41. L. Jiang, F. Schlesinger, C. A. Davis, Y. Zhang, R. Li, M. Salit, T. R. Gingeras, B. Oliver, Synthetic spike-in standards for RNA-seq experiments. *Genome Res.* **21**, 1543–1551 (2011).

42. J. Lovén, D. A. Orlando, A. A. Sigova, C. Y. Lin, P. B. Rahl, C. B. Burge, D. L. Levens, T. I. Lee, R. A. Young, Revisiting global gene expression analysis. *Cell* **151**, 476–482 (2012).

43. M. Bergkessel, D. W. Basta, D. K. Newman, The physiology of growth arrest: Uniting molecular and environmental microbiology. *Nat. Rev. Microbiol.* **14**, 549–562 (2016).

44. M. F. Traxler, S. M. Summers, H.-T. Nguyen, V. M. Zacharia, J. T. Smith, T. Conway, The global, ppGpp-mediated stringent response to amino acid starvation in *Escherichia coli*. *Mol. Microbiol.* **68**, 1128–1148 (2008).

45. T. Durfee, A.-M. Hansen, H. Zhi, F. R. Blattner, D. J. Jin, Transcription profiling of the stringent response in *Escherichia coli*. *J. Bacteriol.* **190**, 1084–1096 (2008).

46. J. Ryals, R. Little, H. Bremer, Control of rRNA and tRNA syntheses in Escherichia coli by guanosine tetraphosphate. *J. Bacteriol.* **151**, 1261–1268 (1982).

47. C. D. Amsler, M. Cho, P. Matsumura, Multiple factors underlying the maximum motility of Escherichia coli as cultures enter post-exponential growth. *J. Bacteriol.* **175**, 6238–6244 (1993).

48. D. Shah, Z. Zhang, A. B. Khodursky, N. Kaldalu, K. Kurg, K. Lewis, Persisters: A distinct physiological state of *E. coli*. *BMC Microbiol.* **6**, 53 (2006).

49. M. Ashburner, C. A. Ball, J. A. Blake, D. Botstein, H. Butler, J. M. Cherry, A. P. Davis, K. Dolinski, S. S. Dwight, J. T. Eppig, M. A. Harris, D. P. Hill, L. Issel-Tarver, A. Kasarskis, S. Lewis, J. C. Matese, J. E. Richardson, M. Ringwald, G. M. Rubin, G. Sherlock, Gene Ontology: Tool for the unification of biology. *Nat. Genet.* **25**, 25–29 (2000).

50. The Gene Ontology Consortium, S. A. Aleksander, J. Balhoff, S. Carbon, J. M. Cherry, H. J. Drabkin, D. Ebert, M. Feuermann, P. Gaudet, N. L. Harris, D. P. Hill, R. Lee, H. Mi, S. Moxon, C. J. Mungall, A. Muruganugan, T. Mushayahama, P. W. Sternberg, P. D. Thomas, K. Van Auken, J. Ramsey, D. A. Siegele, R. L. Chisholm, P. Fey, M. C. Aspromonte, M. V. Nugnes, F. Quaglia, S. Tosatto, M. Giglio, S. Nadendla, G. Antonazzo, H. Attrill, G. dos Santos, S. Marygold, V. Strelets, C. J. Tabone, J. Thurmond, P. Zhou, S. H. Ahmed, P. Asanitthong, D. Luna Buitrago, M. N. Erdol, M. C. Gage, M. Ali Kadhum, K. Y. C. Li, M. Long, A. Michalak, A. Pesala, A. Pritazahra, S. C. C. Saverimuttu, R. Su, K. E. Thurlow, R. C. Lovering, C. Logie, S. Oliferenko, J. Blake, K. Christie, L. Corbani, M. E. Dolan, H. J. Drabkin, D. P. Hill, L. Ni, D. Sitnikov, C. Smith, A. Cuzick, J. Seager, L. Cooper, J. Elser, P. Jaiswal, P. Gupta, P. Jaiswal, S. Naithani, M. Lera-Ramirez, K. Rutherford, V. Wood, J. L. De Pons, M. R. Dwinell, G. T. Hayman, M. L. Kaldunski, A. E. Kwitek, S. J. F. Laulederkind, M. A. Tutaj, M. Vedi, S.-J. Wang, P. D'Eustachio, L. Aimo, K. Axelsen, A. Bridge, N. Hyka-Nouspikel, A. Morgat, S. A. Aleksander, J. M. Cherry, S. R. Engel, K. Karra, S. R. Miyasato, R. S. Nash, M. S. Skrzypek, S. Weng, E. D. Wong, E. Bakker, T. Z. Berardini, L. Reiser, A. Auchincloss, K. Axelsen, G. Argoud-Puy, M.-C. Blatter, E. Boutet, L. Breuza, A. Bridge, C. Casals-Casas, E. Coudert, A. Estreicher, M. Livia Famiglietti, M. Feuermann, A. Gos, N. Gruaz-Gumowski, C. Hulo, N. Hyka-Nouspikel, F. Jungo, P. Le Mercier, D. Lieberherr, P. Masson, A. Morgat, I. Pedruzzi, L. Pourcel, S. Poux, C. Rivoire, S. Sundaram, A. Bateman, E. Bowler-Barnett, H. Bye-A-Jee, P. Denny, A. Ignatchenko, R. Ishtiaq, A. Lock, Y. Lussi, M. Magrane, M. J. Martin, S. Orchard, P. Raposo, E. Speretta, N. Tyagi, K. Warner, R. Zaru, A. D. Diehl, R. Lee, J. Chan, S. Diamantakis, D. Raciti, M. Zarowiecki, M. Fisher, C. James-Zorn, V. Ponferrada, A.

Zorn, S. Ramachandran, L. Ruzicka, M. Westerfield, The Gene Ontology knowledgebase in 2023. *Genetics* **224**, iyad031 (2023).

51. T. Prossliner, K. Gerdes, M. A. Sørensen, K. S. Winther, Hibernation factors directly block ribonucleases from entering the ribosome in response to starvation. *Nucleic Acids Res.* **49**, 2226–2239 (2021).

52. M. Ueta, C. Wada, Y. Bessho, M. Maeda, A. Wada, Ribosomal protein L31 in *Escherichia coli* contributes to ribosome subunit association and translation, whereas short L31 cleaved by protease 7 reduces both activities. *Genes Cells* **22**, 452–471 (2017).

53. C. L. Patten, M. G. Kirchhof, M. R. Schertzberg, R. A. Morton, H. E. Schellhorn, Microarray analysis of RpoS-mediated gene expression in *Escherichia coli* K-12. *Mol. Genet. Genomics* **272**, 580–591 (2004).

54. Y. Shan, A. Brown Gandt, S. E. Rowe, J. P. Deisinger, B. P. Conlon, K. Lewis, ATP-dependent persister formation in *Escherichia coli*. *mBio* **8**, e02267-16 (2017).

55. K. R. Allison, M. P. Brynildsen, J. J. Collins, Metabolite-enabled eradication of bacterial persisters by aminoglycosides. *Nature* **473**, 216–220 (2011).

56. P. W. S. Hill, A. L. Moldoveanu, M. Sargen, S. Ronneau, I. Glegola-Madejska, C. Beetham, R. A. Fisher, S. Helaine, The vulnerable versatility of Salmonella antibiotic persisters during infection. *Cell Host Microbe* **29**, 1757–1773.e10 (2021).

57. M. A. Orman, M. P. Brynildsen, Dormancy is not necessary or sufficient for bacterial persistence. *Antimicrob. Agents Chemother.* **57**, 3230–3239 (2013).

58. M. Schulte, K. Olschewski, M. Hensel, The protected physiological state of intracellular *Salmonella enterica* persisters reduces host cell-imposed stress. *Commun. Biol.* **4**, 520 (2021).

59. G. Manina, N. Dhar, J. D. McKinney, Stress and host immunity amplify mycobacterium tuberculosis phenotypic heterogeneity and induce nongrowing metabolically active forms. *Cell Host Microbe* **17**, 32–46 (2015).

60. O. Braissant, D. Wirz, B. Göpfert, A. U. Daniels, Use of isothermal microcalorimetry to monitor microbial activities. *FEMS Microbiol. Lett.* **303**, 1–8 (2010).

61. R. S. Criddle, A. J. Fontana, D. R. Rank, D. Paige, L. D. Hansen, R. W. Breidenbach, Simultaneous measurement of metabolic heat rate, CO_2 production, and O_2 consumption by microcalorimetry. *Anal. Biochem.* **194**, 413–417 (1991).

62. A. Robador, D. E. LaRowe, S. E. Finkel, J. P. Amend, K. H. Nealson, Changes in microbial energy metabolism measured by nanocalorimetry during growth phase transitions. *Front. Microbiol.* **9**, 109 (2018).

63. L. Grassi, M. Di Luca, G. Maisetta, A. C. Rinaldi, S. Esin, A. Trampuz, G. Batoni, Generation of persister cells of *Pseudomonas aeruginosa* and *Staphylococcus aureus* by chemical treatment and evaluation of their susceptibility to membrane-targeting agents. *Front. Microbiol.* **8**, 1917 (2017).

64. E. Biselli, S. J. Schink, U. Gerland, Slower growth of *Escherichia coli* leads to longer survival in carbon starvation due to a decrease in the maintenance rate. *Mol. Syst. Biol.* **16**, e9478 (2020).

65. M. I. Love, W. Huber, S. Anders, Moderated estimation of fold change and dispersion for RNA-seq data with DESeq2. *Genome Biol.* **15**, 550 (2014).

66. A. C. St John, A. L. Goldberg, Effects of reduced energy production on protein degradation, guanosine tetraphosphate, and RNA synthesis in *Escherichia coli*. *J. Biol. Chem.* **253**, 2705–2711 (1978).

67. D. Nguyen, A. Joshi-Datar, F. Lepine, E. Bauerle, O. Olakanmi, K. Beer, G. McKay, R. Siehnel, J. Schafhauser, Y. Wang, B. E. Britigan, P. K. Singh, Active starvation responses mediate antibiotic tolerance in biofilms and nutrient-limited bacteria. *Science* **334**, 982–986 (2011).

68. S. Schink, C. Ammar, Y.-F. Chang, R. Zimmer, M. Basan, Analysis of proteome adaptation reveals a key role of the bacterial envelope in starvation survival. *Mol. Syst. Biol.* **18**, e11160 (2022).

69. L. Boulos, M. Prévost, B. Barbeau, J. Coallier, R. Desjardins, LIVE/DEAD BacLight: Application of a new rapid staining method for direct enumeration of viable and total bacteria in drinking water. *J. Microbiol. Methods* **37**, 77–86 (1999).

70. H. M. Davey, P. Hexley, Red but not dead? Membranes of stressed *Saccharomyces cerevisiae* are permeable to propidium iodide. *Environ. Microbiol.* **13**, 163–171 (2011).

71. Y. Yang, O. Karin, A. Mayo, X. Song, P. Chen, A. L. Santos, A. B. Lindner, U. Alon, Damage dynamics and the role of chance in the timing of *E. coli* cell death. *Nat. Commun.* **14**, 2209 (2023).

72. M. Kohram, A. E. Sanderson, A. Loui, P. V. Thompson, H. Vashistha, A. Shomar, Z. N. Oltvai, H. Salman, Nonlethal deleterious mutation–induced stress accelerates bacterial aging. *Proc. Natl. Acad. Sci. U.S.A.* **121**, e2316271121 (2024).

73. M. G. Harrington, The action of chloramphenicol on protein and nucleic acid synthesis by Escherichia coli strain B. *Microbiology* **18**, 767–773 (1958).

74. A. Manten, M. J. Wisse, Antagonism between antibacterial drugs. *Nature* **192**, 671–672 (1961).

75. D. H. de Groot, A. J. Tjalma, F. J. Bruggeman, E. van Nimwegen, Effective bet-hedging through growth rate dependent stability. *Proc. Natl. Acad. Sci. U.S.A.* **120**, e2211091120 (2023).

76. W. K. Smits, O. P. Kuipers, J.-W. Veening, Phenotypic variation in bacteria: The role of feedback regulation. *Nat. Rev. Microbiol.* **4**, 259–271 (2006).

77. D. Choudhary, V. Lagage, K. R. Foster, S. Uphoff, Phenotypic heterogeneity in the bacterial oxidative stress response is driven by cell-cell interactions. *Cell Rep.* **42**, 112168 (2023).

78. W. W. K. Mok, J. O. Park, J. D. Rabinowitz, M. P. Brynildsen, RNA futile cycling in model persisters derived from MazF accumulation. *mBio* **6**, e01588-15 (2015).

79. J. Errington, Regulation of endospore formation in *Bacillus subtilis*. *Nat. Rev. Microbiol.* **1**, 117–126 (2003).

80. E. Segev, A. Rosenberg, G. Mamou, L. Sinai, S. Ben-Yehuda, Molecular kinetics of reviving bacterial spores. *J. Bacteriol.* **195**, 1875–1882 (2013).

81. J. C. Vary, H. O. Halvorson, Kinetics of germination of *Bacillus* spores. *J. Bacteriol.* **89**, 1340–1347 (1965).

82. O. Fridman, A. Goldberg, I. Ronin, N. Shoresh, N. Q. Balaban, Optimization of lag time underlies antibiotic tolerance in evolved bacterial populations. *Nature* **513**, 418–421 (2014).

83. Y. Shan, D. Lazinski, S. Rowe, A. Camilli, K. Lewis, Genetic basis of persister tolerance to aminoglycosides in *Escherichia coli*. *mBio* **6**, e00078-15 (2015).

84. H. S. Girgis, A. K. Hottes, S. Tavazoie, Genetic architecture of intrinsic antibiotic susceptibility. *PLOS ONE* **4**, e5629 (2009).

85. H. Luidalepp, A. Jõers, N. Kaldalu, T. Tenson, Age of inoculum strongly influences persister frequency and can mask effects of mutations implicated in altered persistence. *J. Bacteriol.* **193**, 3598–3605 (2011).

86. P. J. T. Johnson, B. R. Levin, Pharmacodynamics, population dynamics, and the evolution of persistence in *Staphylococcus aureus*. *PLOS Genet.* **9**, e1003123 (2013).

87. N. Vázquez-Laslop, H. Lee, A. A. Neyfakh, Increased persistence in *Escherichia coli* caused by controlled expression of toxins or other unrelated proteins. *J. Bacteriol.* **188**, 3494–3497 (2006).

88. S. V. Sharma, D. Y. Lee, B. Li, M. P. Quinlan, F. Takahashi, S. Maheswaran, U. McDermott, N. Azizian, L. Zou, M. A. Fischbach, K.-K. Wong, K. Brandstetter, B. Wittner, S. Ramaswamy, M. Classon, J. Settleman, A chromatin-mediated reversible drug-tolerant state in cancer cell subpopulations. *Cell* **141**, 69–80 (2010).

89. D. K. Singh, C.-J. Ku, C. Wichaidit, R. J. Steininger III, L. F. Wu, S. J. Altschuler, Patterns of basal signaling heterogeneity can distinguish cellular populations with different drug sensitivities. *Mol. Syst. Biol.* **6**, 369 (2010).

90. M. B. Elowitz, A. J. Levine, E. D. Siggia, P. S. Swain, Stochastic gene expression in a single cell. *Science* **297**, 1183–1186 (2002).

91. J. S. Wolfson, D. C. Hooper, G. L. McHugh, M. A. Bozza, M. N. Swartz, Mutants of Escherichia coli K-12 exhibiting reduced killing by both quinolone and beta-lactam antimicrobial agents. *Antimicrob. Agents Chemother.* **34**, 1938–1943 (1990).

92. R. Lutz, H. Bujard, Independent and tight regulation of transcriptional units in *Escherichia coli* via the LacR/O, the TetR/O and AraC/I1-I2 regulatory elements. *Nucleic Acids Res.* **25**, 1203–1210 (1997).

93. S. Metzger, G. Schreiber, E. Aizenman, M. Cashel, G. Glaser, Characterization of the *relA1* mutation and a comparison of *relA1* with new *relA* null alleles in *Escherichia coli*. *J. Biol. Chem.* **264**, 21146–21152 (1989).

94. N. P. Fiil, B. M. Willumsen, J. D. Friesen, K. von Meyenburg, Interaction of alleles of therelA, relC andspoT genes in *Escherichia coli*: Analysis of the interconversion of GTP, ppGpp and pppGpp. *Mol. Gen. Genet.* **150**, 87–101 (1977).

95. T. Durfee, R. Nelson, S. Baldwin, G. Plunkett III, V. Burland, B. Mau, J. F. Petrosino, X. Qin, D. M. Muzny, M. Ayele, R. A. Gibbs, B. Csörgő, G. Pósfai, G. M. Weinstock, F. R. Blattner, The complete genome sequence of *Escherichia coli* DH10B: Insights into the biology of a laboratory workhorse. *J. Bacteriol.* **190**, 2597–2606 (2008).

96. O. Gefen, C. Gabay, M. Mumcuoglu, G. Engel, N. Q. Balaban, Single-cell protein induction dynamics reveals a period of vulnerability to antibiotics in persister bacteria. *Proc. Natl. Acad. Sci. U.S.A.* **105**, 6145–6149 (2008).

97. E. Rotem, A. Loinger, I. Ronin, I. Levin-Reisman, C. Gabay, N. Shoresh, O. Biham, N. Q. Balaban, Regulation of phenotypic variability by a threshold-based mechanism underlies bacterial persistence. *Proc. Natl. Acad. Sci. U.S.A.* **107**, 12541–12546 (2010).

98. K. A. Datsenko, B. L. Wanner, One-step inactivation of chromosomal genes in Escherichia coli K-12 using PCR products. *Proc. Natl. Acad. Sci. U.S.A.* **97**, 6640–6645 (2000).

99. I. Levin-Reisman, O. Gefen, O. Fridman, I. Ronin, D. Shwa, H. Sheftel, N. Q. Balaban, Automated imaging with ScanLag reveals previously undetectable bacterial growth phenotypes. *Nat. Methods* **7**, 737–739 (2010).

100. T. Tosa, L. I. Pizer, Effect of serine hydroxamate on the growth of *Escherichia coli*. *J. Bacteriol.* **106**, 966–971 (1971).

101. A. D. Edelstein, M. A. Tsuchida, N. Amodaj, H. Pinkard, R. D. Vale, N. Stuurman, Advanced methods of microscope control using μManager software. *J. Biol. Methods* **1**, e10 (2014).

102. L. Potvin-Trottier, S. Luro, J. Paulsson, Microfluidics and single-cell microscopy to study stochastic processes in bacteria. *Curr. Opin. Microbiol.* **43**, 186–192 (2018).

103. J. Schindelin, I. Arganda-Carreras, E. Frise, V. Kaynig, M. Longair, T. Pietzsch, S. Preibisch, C. Rueden, S. Saalfeld, B. Schmid, J.-Y. Tinevez, D. J. White, V. Hartenstein, K. Eliceiri, P. Tomancak, A. Cardona, Fiji: An open-source platform for biological-image analysis. *Nat. Methods* **9**, 676–682 (2012).

104. M. Martin, Cutadapt removes adapter sequences from high-throughput sequencing reads. *EMBnet J.* **17**, 10 (2011).

105. B. Langmead, S. L. Salzberg, Fast gapped-read alignment with Bowtie 2. *Nat. Methods* **9**, 357–359 (2012).

106. S. Anders, P. T. Pyl, W. Huber, HTSeq—A Python framework to work with high-throughput sequencing data. *Bioinformatics* **31**, 166–169 (2015).

107. B. T. Sherman, M. Hao, J. Qiu, X. Jiao, M. W. Baseler, H. C. Lane, T. Imamichi, W. Chang, DAVID: A web server for functional enrichment analysis and functional annotation of gene lists (2021 update). *Nucleic Acids Res.* **50**, W216–W221 (2022).

108. D. W. Huang, B. T. Sherman, R. A. Lempicki, Systematic and integrative analysis of large gene lists using DAVID bioinformatics resources. *Nat. Protoc.* **4**, 44–57 (2009).

109. J. Suurkuusk, M. Suurkuusk, P. Vikegard, A multichannel microcalorimetric system. *J. Therm. Anal. Calorim.* **131**, 1949–1966 (2018).

110. T. Kamada, S. Kawai, An algorithm for drawing general undirected graphs. *Inf. Process. Lett.* **31**, 7–15 (1989).

111. T. Prossliner, K. S. Winther, M. A. Sørensen, K. Gerdes, Ribosome hibernation. *Annu. Rev. Genet.* **52**, 321–348 (2018).

112. S. Lilleorg, K. Reier, A. Pulk, A. Liiv, T. Tammsalu, L. Peil, J. H. D. Cate, J. Remme, Bacterial ribosome heterogeneity: Changes in ribosomal protein composition during transition into stationary growth phase. *Biochimie* **156**, 169–180 (2019).

113. T. Minamino, N. Terahara, S. Kojima, K. Namba, Autonomous control mechanism of stator assembly in the bacterial flagellar motor in response to changes in the environment. *Mol. Microbiol.* **109**, 723–734 (2018).

114. S. Guo, J. Liu, The bacterial flagellar motor: Insights into torque generation, rotational switching, and mechanosensing. *Front. Microbiol.* **13**, 911114 (2022).

115. G. H. Wadhams, J. P. Armitage, Making sense of it all: Bacterial chemotaxis. *Nat. Rev. Mol. Cell Biol.* **5**, 1024–1037 (2004).
